# Supplementary material for: Adolescent resilience and mobile phone addiction in Henan Province of China: Impacts of chain mediating, coping style
Source: PLoS One. 2022 Dec 27;17(12):e0278182. doi: 10.1371/journal.pone.0278182 (PMC9794036; doi:10.1371/journal.pone.0278182)
Supplement: Attachment — Submitted filename: PONE-D-22-09528_reviewer_tcsw01.pdf [file pone.0278182.s002.pdf]

PLOS ONE

Adolescent Resilience and Mobile Phone Addiction in Henan province of China: Impacts of Chain Mediating, Coping Style

--Manuscript Draft--

**Manuscript Number:**	PONE-D-22-09528
**Article Type:**	Research Article
**Full Title:**	Adolescent Resilience and Mobile Phone Addiction in Henan province of China: Impacts of Chain Mediating, Coping Style
**Short Title:**	The relationship between psychological resilience and mobile phone addiction in adolescents
**Corresponding Author:**	Anna Ma<br>Xinxiang Medical University<br>Xinxiang, CHINA
**Keywords:**	Keywords: Adolescent resilience; Coping style; Mobile phone addiction; China; DASS-21; Chain mediating
**Abstract:**	Abstract<br>Background: As mobile phone use grows, so it brings benefits and risks. As an important part of adolescents healthy growth, resilience plays an indispensable role in Chinese adolescents. Thus, it is important to identify when mobile phone of adolescent use is addiction. This study proposed to explore the effects of adolescent resilience on mobile phone addiction, and tested the mediating role of coping style and depression, anxiety, and stress (DASS) on phone addiction among 2268 adolescents in the Henan Province.<br>Methods: The adolescents were surveyed via an online questionnaire, and we used structural equation modeling to examine the correlations and moderation effects. All data analyses were performed using SPSS 26.0 and Amos 23.0.<br>Results: The results show that both coping style and DASS could mediate the relationship between adolescent resilience and mobile phone addiction among Chinese adolescents. The relationship between adolescent resilience and mobile phone addiction in Chinese adolescents was mediated by the chain of coping styles and DASS.<br>Conclusions: There is a negative relationship exists between resilience and mobile phone addiction in this population. In addition, stress, anxiety, depression, and coping style significantly influence the risk of adolescent mobile phone addiction and play an intermediary role in Chinese adolescent resilience and mobile phone addiction.
**Order of Authors:**	Anna Ma
	Hongjuan Chang
**Additional Information:**	
**Question**	**Response**
**Financial Disclosure**<br><br>Enter a financial disclosure statement that describes the sources of funding for the work included in this submission. Review the submission guidelines for detailed requirements. View published research articles from *PLOS ONE* for specific examples.<br><br>This statement is required for submission and **will appear in the published article** if the submission is accepted. Please make	The funders had no role in study design, data collection and analysis, decision to publish, or preparation of the manuscript.

sure it is accurate.<br><br>**Unfunded studies**<br>Enter: *The author(s) received no specific funding for this work.*<br><br>**Funded studies**<br>Enter a statement with the following details:<br>• Initials of the authors who received each award<br>• Grant numbers awarded to each author<br>• The full name of each funder<br>• URL of each funder website<br>• Did the sponsors or funders play any role in the study design, data collection and analysis, decision to publish, or preparation of the manuscript?<br>• **NO** - Include this sentence at the end of your statement: *The funders had no role in study design, data collection and analysis, decision to publish, or preparation of the manuscript.*<br>• **YES** - Specify the role(s) played.<br><br>* typeset	
**Competing Interests**<br><br>Use the instructions below to enter a competing interest statement for this submission. On behalf of all authors, disclose any competing interests that could be perceived to bias this work—acknowledging all financial support and any other relevant financial or non-financial competing interests.<br><br>This statement is required for submission and **will appear in the published article** if the submission is accepted. Please make sure it is accurate and that any funding sources listed in your Funding Information later in the submission form are also declared in your Financial Disclosure statement.<br><br>View published research articles from *PLOS ONE* for specific examples.	The authors have declared that no competing interests exist.

**NO authors have competing interests**<br><br>Enter: *The authors have declared that no competing interests exist.*<br><br>**Authors with competing interests**<br><br>Enter competing interest details beginning with this statement:<br><br>*I have read the journal's policy and the authors of this manuscript have the following competing interests: [insert competing interests here]*<br><br>* typeset	
**Ethics Statement**<br><br>Enter an ethics statement for this submission. This statement is required if the study involved:<br><br>• Human participants<br>• Human specimens or tissue<br>• Vertebrate animals or cephalopods<br>• Vertebrate embryos or tissues<br>• Field research<br><br>Write "N/A" if the submission does not require an ethics statement.<br><br>General guidance is provided below. Consult the submission guidelines for detailed instructions. **Make sure that all information entered here is included in the Methods section of the manuscript.**	The participants were under 18 years old, and we provided informed consent to their parents (or legal guardians). All procedures performed in studies involving human participants were approved by the Ethics Committee of Xinxiang Medical University(#XYLL-2018015).

Powered by Editorial Manager® and ProduXion Manager® from Aries Systems Corporation

**Format for specific study types**<br><br>**Human Subject Research (involving human participants and/or tissue)**<br>• Give the name of the institutional review board or ethics committee that approved the study<br>• Include the approval number and/or a statement indicating approval of this research<br>• Indicate the form of consent obtained (written/oral) or the reason that consent was not obtained (e.g. the data were analyzed anonymously)<br><br>**Animal Research (involving vertebrate animals, embryos or tissues)**<br>• Provide the name of the Institutional Animal Care and Use Committee (IACUC) or other relevant ethics board that reviewed the study protocol, and indicate whether they approved this research or granted a formal waiver of ethical approval<br>• Include an approval number if one was obtained<br>• If the study involved *non-human primates*, add *additional details* about animal welfare and steps taken to ameliorate suffering<br>• If anesthesia, euthanasia, or any kind of animal sacrifice is part of the study, include briefly which substances and/or methods were applied<br><br>**Field Research**<br><br>Include the following details if this study involves the collection of plant, animal, or other materials from a natural setting:<br>• Field permit number<br>• Name of the institution or relevant body that granted permission	
**Data Availability**<br><br>Authors are required to make all data underlying the findings described fully available, without restriction, and from the time of publication. PLOS allows rare exceptions to address legal and ethical concerns. See the PLOS Data Policy and FAQ for detailed information.	No - some restrictions will apply

A Data Availability Statement describing where the data can be found is required at submission. Your answers to this question constitute the Data Availability Statement and **will be published in the article**, if accepted.<br><br>**Important:** Stating 'data available on request from the author' is not sufficient. If your data are only available upon request, select 'No' for the first question and explain your exceptional situation in the text box.<br><br>Do the authors confirm that all data underlying the findings described in their manuscript are fully available without restriction?	
**Describe where the data may be found in full sentences. If you are copying our sample text, replace any instances of XXX with the appropriate details.**<br><br>• If the data are **held or will be held in a public repository**, include URLs, accession numbers or DOIs. If this information will only be available after acceptance, indicate this by ticking the box below. For example: *All XXX files are available from the XXX database (accession number(s) XXX, XXX.*).<br>• If the data are all contained **within the manuscript and/or Supporting Information files**, enter the following: *All relevant data are within the manuscript and its Supporting Information files.*<br>• If neither of these applies but you are able to provide **details of access elsewhere**, with or without limitations, please do so. For example:<br><br>*Data cannot be shared publicly because of [XXX]. Data are available from the XXX Institutional Data Access / Ethics Committee (contact via XXX) for researchers who meet the criteria for access to confidential data.*<br><br>*The data underlying the results presented in the study are available from (include the name of the third party*	Due to the potential for indirect adolescents and their parents recognition of this adolescent subgroup, the datasets generated during the current study are not publicly available but are available from the corresponding author on reasonable request.

Powered by Editorial Manager® and ProduXion Manager® from Aries Systems Corporation

and contact information or URL).<br>• This text is appropriate if the data are owned by a third party and authors do not have permission to share the data.<br><br>* typeset	
Additional data availability information:	

Click here to access/download;Manuscript;Adolescent Resilience and Mobile Phone Addiction.docx

Adolescent Resilience and Mobile Phone Addiction in Henan province of China: Impacts of Chain Mediating, Coping Style

Anna Ma[1 #a #b], Yan Yang[1], Shuangxi Guo[2], Xue Li[1], Shenhua Zhang[3], Hongjuan Chang[1*]

[1 #a] Xinxiang Medical University ,School of Nursing, Xinxiang, Henan province,China

[#b] St. Paul University, School of Nursing, Manila, Philippines

[2]The First Affiliated Hospital of Xinxiang Medical University, Department of Neurology, Xinxiang, Henan province, China

[3] Weihui Senior Middle School, Xinxiang, China

*** Correspondence:**

Hong juan Chang

E-mail: changhj0812@126.com

Conflict of Interest/Disclosure Statement

Competing interests

The authors declare that they have no competing interests and all methods were carried out in accordance with relevant guidelines and regulations. The authors alone are responsible for the content and writing of the article.

Funding

This work was supported by the National Natural Science Foundation of China(grant number: 81803252).

Ethics approval and consent to participate

The participants were under 18 years old, and we provided informed consent to their parents (or legal guardians). All procedures performed in studies involving human participants were approved by the Ethics Committee of Xinxiang Medical University(#XYLL-2018015).

Acknowledgements

Not applicable.

Abstract

Background: As mobile phone use grows, so it brings benefits and risks. As an important part of adolescents healthy growth, resilience plays an indispensable role in Chinese adolescents. Thus, it is important to identify when mobile phone of adolescent use is addiction. This study proposed to explore the effects of adolescent resilience on mobile phone addiction, and tested the mediating role of coping style and depression, anxiety, and stress (DASS) on phone addiction among 2268 adolescents in the Henan Province.

Methods: The adolescents were surveyed via an online questionnaire, and we used structural equation modeling to examine the correlations and moderation effects. All data analyses were performed using SPSS 26.0 and Amos 23.0.

Results: The results show that both coping style and DASS could mediate the relationship between adolescent resilience and mobile phone addiction among Chinese adolescents. The relationship between adolescent resilience and mobile phone addiction in Chinese adolescents was mediated by the chain of coping styles and DASS.

Conclusions: There is a negative relationship exists between resilience and mobile phone addiction in this population. In addition, stress, anxiety, depression, and coping style significantly influence the risk of adolescent mobile phone addiction and play an intermediary role in Chinese adolescent resilience and mobile phone addiction.

Keywords: Adolescent resilience; Coping style; Mobile phone addiction; China; DASS-21; Chain mediating

Introduction

With the development of information technology, the functions of mobile phone is becoming more and more powerful, it is considered as an important communication tool and social accessory. Due to the COVID-19 outbreak, mobile phones have become increasingly important for online teaching and learning in China. According to the 48th China statistical report on internet development, by June 2021, there were 1.07 billion mobile phone users in China, accounting for 99.7% of the total number of internet users. Furthermore, internet users spend an average of 26.9 hours online per week, and the number of internet users between the ages of 6 and 19 reached 158 million, accounting for 15.7% of the total(China Internet Network Information Center,2021). As part of normal adolescent psychological development, this age group develops susceptibility to peer influences and tends to have low-risk perception—factors that can result in increased risk-taking behavior and poor self-regulation (Patton GC,2016). Regarding the emotional state of adolescents, stressful life events have been identified as a significant risk factor, and the spread of COVID-19 led to substantial social and economic changes because many governments adapted quarantine policies to assure a population of safety and prevent the virus from spreading. Psychopathologists focused on the psychological impact of COVID-19 and its variations in the adolescent population; they identified moderate to severe levels of stress, anxiety, and depression in this population (Ozamiz-Etxebarria, N.,2020). There was a moderate positive correlation between negative coping style and adolescents' mobile phone addiction

(Guang Li Lu,2021).

In the middle of July 2021, the Henan province suffered from unusually heavy rainfall and maximum continuous rainfall of 958 mm (The Tenth Press Conference of Henan Province Flood Control and Disaster Relief,2021), causing severe flooding. The flood, named the “7.20 Henan rainstorm,” blasted the overwhelmed dams and banks of rivers in a short time, causing severe traffic paralysis, water power failure, waterlogging, and upending tens of millions of lives. Randeniya reported that sleeping difficulties were the most affected problem by flooding disasters in adolescents of Sri Lanka (Enoka Randeniya,2018). Makwana indicated that the psychological effects of the disaster were fiercer among children, women, and the dependent elderly population (Makwana N,2019). Furthermore, research has shown significant differences exist in psychological effects among adolescents in terms of gender and family size(Satadeepa Som,2019; Breik,2019).

Many scholars believe that adolescent resilience is also related to substance use, such as smoking and excessive drinking (alcoholism) (Davis SJ,2011). Some experts also believe that depression, anxiety, and pressure may lead to internet addiction(Carli V,2013). However, studies on the relationship between mental resilience and adolescents’ coping styles, mental health, and mobile phone addiction are rare.

In view of this, adolescents confined to their homes may be likely to overuse mobile phones and the internet due to floods in Henan province of China. Thus, we hypothesized that there is a correlation between resilience and mobile phone addiction among adolescents and that coping style and mental health play a mediating role in that

relationship.

“Coping” means constantly changing cognitive and behavioral efforts to manage specific external and/or internal demands that are appraised as taxing or exceeding a person’s resources (Lazarus, R. S.,1984). Chapman thought that adolescents with lower self-esteem engage in coping strategies of ventilating feelings, avoiding problems, and relaxing, and adolescents with higher self-esteem were more likely to engage in coping styles that directly address solving the problem(Chapman, Paula L.1999). During the COVID-19 pandemic, children who used positive strategies to cope with the situation suffered less emotionally and behaviorally (Orgilés M,2021). Fang Liu found negative coping style mediated the relationship between smartphone addiction and childhood psychological maltreatment(Fang Liu,2020). Psychological resilience is the ability to cope with a crisis or to quickly return to a pre-crisis status from mentally or emotionally (de Terte, I., & Stephens, C,2014). Resilience is an important developmental stage during adolescence, as it is a transitional period characterized by significant neurobiological and psychosocial changes in the context of amplifying environmental demands and increasing sensitivity to social contexts (Schriber, R. A. & Guyer, A. E,2016).

Resilience has neurobiological, physical, social, cognitive-behavioral, and emotional regulation foundations (Southwick, S. M.,2014; Southwick, S. M. & Charney, D. S,2012; Hu, T., Zhang, D. & Wang, J,2015). However, how the neurobiological and psychosocial factors influence resilience in adolescence is not well understood. Malhi et al. proposed a model that captures the dynamic nature of resilience in adolescents,

with special attention to how it emerges, how neurobiological and psychosocial factors interact to build and strengthen resilience, and individual differences in resilience (Malhi, G.S., 2019). Resilience negatively predicted the negative coping style and positively predicted the positive coping style, life events not only had direct influenced negative coping style and positive coping style but also had indirect influenced coping styles by affecting resilience(Li J,2020). The mutually enhancing relationship between resilience and positive mental health, and vice versa, a mutually reducing relationship between resilience and mental illness, and presented the significant influence of mental health level on resilience(Wu Y,2020). Malek showed that avoidant coping styles can aggravate depressive, anxiety, and stress symptoms in participants during the COVID-19 pandemic. Keeping optimism, resilience, and approach coping styles can decrease the mental health burden of the pandemic on participants (Smida, M.,2021).

Based on the literature review above, the present study constructed a chain mediation model to examine the mediating role of negative coping, stress, anxiety, and depression in the relationship between adolescent resilience and mobile phone addiction among Chinese adolescents. Furthermore, we proposed a model to test the associations among Chinese adolescent resilience, coping style, mental health, and mobile phone addiction, to further clarify mobile phone addiction related to resilience (Figure 1).

Methods

Participants

The convenience sampling method was employed to select students from grades 7th to 9th of middle school and grades 1st to 3rd of high school in Henan province of China to complete the questionnaires online from July 1 to August30, 2021. In total, 2268 valid questionnaires were obtained, with an effective rate of 97.28%. Among them, the mean age was 14.90 years (*SD* = 2.58, range = 12–21 years), participants included 979 boys(43.20%)and 1289 girls (56.80%). They completed a survey that included demographic variables, a mobile phone addiction index (MPAI), a depression, anxiety, and stress scale with 21 items (DASS-21), the Resilience Scale for Chinese Adolescents (RSCA), and the Simplified coping style questionnaire (SCSQ).

Measurement of Structures

DASS-21

The DASS-21was used to evaluate negative emotional states of depression, anxiety, and stress(Lovibond, P.F., Lovibond, S.H,1995), refer to the previous week, and each item is classified into four Likert responses from 0 to 3, from 0 = “nothing” to 3 = “Most of the time.” This self-report instrument includes three subscales: 1) the stress subscale, which measures tension, agitation, difficulty relaxing, and negative affection; 2)the anxiety subscale, which assesses autonomic arousal, skeletal musculature effects, situational anxiety, and subjective experience of anxious arousal; and 3) the depression subscale, which measures hopelessness, dysphoria, lack of interest, self-deprecation, and inertia. The reliability coefficients of depression,

anxiety and stress were 0.82, 0.82 and 0.79, respectively. The Cronbach's alpha of the total scale was 0.89.

MPAI

The MPAI was designed by Louis Leung to identify addiction symptoms associated with mobile phone use among adolescents in Hong Kong(Louis Leung,2008). The scale includes 17 items answered on a five-point Likert scale of 1 to 5 (1 = not at all; 2 = rarely; 3 = occasionally; 4 = often; and 5 = always). The scale covers four dimensions: 1) "inability to control craving," which reflects the amount of time adolescents spend on the mobile phone, thereby leading to complaints from family and friends about their compulsive mobile phone use and causing the adolescents loss of sleep due to the excessive use; 2) "Anxiety and feeling lost" assesses preoccupation, feeling lost or anxious, and having difficulty switching off the mobile phone; 3)"productivity loss" measures decreased productivity and diverted attention from pressing issues due to adolescents' excessive use mobile phones; 4) "withdrawal and escape" indicates that adolescents use their mobile phones to escape from isolation, loneliness, and feeling down. The Cronbach's alpha of scale was 0.90.

RSCA

The RSCA was developed by Yueqin Hu(Yueqin Hu, 2008)according to the process model of the resilience concept and applied to Chinese adolescents. There are 27 items divided into two factors: "manpower" and "support"; the former includes three factors: goal focus, emotion control, and positive cognition; the latter includes two

factors: family support and interpersonal assistance. The reliability of the total scale was 0.85.

SCSQ

This SCSQ was designed by Ya-Ning Xie (Ya-Ning Xie,1998), combined with the characteristics of culture in China, to simplify the ways of coping questionaires and modify, compiled a simple coping style questionnaire, composed of 20 items, often involving people in daily life may take different attitude and measures, according to people in relatively separated suffered setbacks coping styles can be two categories: “positive coping” and “negative coping." The reliability of the total scale was 0.90, while the positive coping and negative response subscales were 0.89 and 0.78, respectively.

Data Analysis

All data analyses were performed using SPSS 26.0 and Amos 23 (IBM Inc., Armonk, NY, USA). First, descriptive data were received using SPSS 26.0, and correlations variables were calculated using Pearson’s correlations. Second, according to Baron and Kenny(Baron,R.M.,Kenny,D.A. ,1986), we analyzed the mediation effects using two measurement models to examine how well the indicators represented each latent variable. Second, we tested the hypothesized relationships among latent variables. Maximum likelihood (ML) estimation was used to test the two structural models in the AMOS 23.0 program. When TLI > 0.90, CFI > 0.90, and RMSEA < 0.06, the

model fits well, according to Hu and Bentler (Hu, L. T., 1999).We followed the stepwise method to structure the best-fitting model for the mediated effects and bootstrapping with 5000 replications to measure the chain mediation model. All data analyses were two-tailed, with significance levels of $P < 0.01$ and $P < 0.05$.

Results

Descriptive Statistics

We included 2268 participants, including 979 boys (43.20%) and 1289 girls (56.80%), in the final analysis. The proportion of girls was slightly higher than that of boys (56.80% vs. 43.20%). The mean age was 14.90 years (SD = 2.58, range 12–21 years). There were 1244 (54.85%) participants in the middle school, 1,024 (45.15%) in the high school, 169 (7.50%) from one-child families, 2099 (92.50%) were from non-one-child families. The other results are shown in Table 1.

Univariate Analysis

As displayed in Table 2, the results for the 2268 participants, The category totals as total mean (*SD*) are as follows: MPAI, 39.57 (±13.82); DASS-21, 5.190 (±4.57); positive coping was 22.45 (±9.18); negative coping, 11.93 (±5.64); RSCA, 88.89 (±18.50).

Correlation Analysis of Major Study Variables

The variables correlated with the constructs in Table 3 were less than 0.85. The discriminant validity value (< 0.85) was met in the construct correlation (Kline RB,2005). These findings showed that valid and reliable constructs were used.

Structural Model Testing and Structural Relationship Between Constructs

The test results revealed the goodness of fit of the proposed structural model ($\chi 2/df$ = 2.57, RMSEA = 0.054, GFI = 0.978, CFI = 0.984). The hypothesis relationships between the variates are demonstrated in Table 4 and Figure 2. The indirect effects are presented in Table 5. Bootstrapping (the process was repeated 5000 times)analyses showed that the indirect effects of adolescent resilience on mobile phone addiction through negative coping and stress, anxiety, and depression were significant and positive (standardized indirect effect 0.029,95%CI [0.012,0.048], $P < 0.01$), and the indirect effect of adolescent resilience on mobile phone addiction through stress, anxiety, depression was 0.111, 95% CI [0.045, 0.186], $P < 0.01$, excluding 0, and mediating effect was significant. The indirect effect of adolescent resilience on mobile phone addiction through negative coping was -0.092, 95% CI [-0.125, -0.061], $P < 0.01$, excluding 0, and the mediating effect was significant.

Discussion

This study surveyed the ways by which adolescent psychological resilience, coping style and Depression, anxiety, and stress affected mobile phone addiction among Chinese adolescents. The results showed that adolescent psychological resilience

could directly and negatively affect mobile phone addiction in Chinese adolescents, which is consistent with previous research findings (Robertson, T.W., 2018). According to the psychological resilience framework theory (Kumpfe K.L. , 2004), psychological resilience is an important protective factor for problem behavior and personal mental health. Griffiths argued that addictions consist of several components, such as relapse, mood modification, tolerance, conflict, and withdrawal (Mark Griffiths,2005). In the studies, psychological resilience was found to protect the addictive behaviors (internet problematic use)(Lee Y.K.,2014; Hou X.L.,2017).Adolescent mobile phone addiction affect their life and study, and this study suggests that family, peer, teacher support, and exercise help enhance the brain and make it more resilient to adversity and stress.

The experimental results show that negative coping style is negatively correlated with adolescents psychological resilience, negative coping style is negatively correlated with mobile phone addiction. It shows that adolescents with good psychological resilience are more likely to adopt positive coping styles when facing pressure and frustration and are less likely to addicted to mobile phones; this results show that negative coping styles and depression, anxiety, and stress (DASS) play intermediary roles, respectively, in adolescent resilience and mobile phone addiction in Chinese adolescents; thus, our hypothesis was verified. This finding is consistent with the results of previous studies. Understanding how one copes with stress and managing coping styles can be particularly effective for smartphone addiction (Alan R, 2020).

The Simplified Coping Style Questionnaire(SCSQ) results revealed that coping style had a maximal effect on adolescent mobile phone addiction (Lu GL,2021). Many studies have indicated a relationship between depression, anxiety, and loneliness with smartphone usage (Ozen S, 2017; Elhai JD,2018). Depression and social anxiety are risk determinants for greater problematic smartphone use (Pera A,2020). Stress, anxiety, and depression were significantly positively correlated with smartphone addiction (Sonali Tanmay Choksi, 2021). The found a significant positive relationship between anxiety about COVID-19 infection and daily smartphone use hours; the largest predictor of smartphone addiction was anxiety about COVID-19 infection (Al. Qudah, M.F.,2021).

Negative coping style and DASS played a continuous intermediary role in the impact of adolescent resilience on mobile phone addiction among Chinese adolescents. Smartphone users who experience depressive symptoms may similarly use their mobile devices as a coping strategy to alleviate these unpleasant symptoms (Ahn, S. Y., 2015). Coping and affective disorders appear to play key roles in international addiction among adolescents (Einar B,2017). COVID-19 and floods as stressors can cause psychological stress responses in adolescents, and differences in coping styles can cause differences in behaviors in adolescents. Coping style is a significant factor leading to smartphone addiction among adolescents. Problem-focused coping strategies indicate that coping behaviors directly target the source of stress and can prompt participants to use positive coping styles to deal with the adverse

consequences of the pandemic; conversely, coping styles of avoidance, denial, and fantasy in dealing with stress make it a potentially strong risk factor for smartphone addiction (Stahl GK, 2005; Duan L,2021).

Limitations

This study had several limitations. The convenience sample limits the universality of the results. Factors such as family environment, personality traits, peer relationships, and sleep quality may also affect mobile phone addiction among adolescents. Therefore, future studies should examine whether the relationship between Chinese adolescent resilience, coping style, DASS, and mobile phone addiction will change over time.

Conclusions

This study explored the impact mechanism of the effect of resilience on mobile phone addiction among Chinese adolescents during a pandemic and flood. The structural equation model was utilized to synchronously examine the individual and continuous mediating roles of coping styles and DASS. This study results indicate a negative relationship exists between resilience and mobile phone addiction in this population. In addition, stress, anxiety, depression, and coping style significantly influence the risk of adolescent mobile phone addiction and play an intermediary role in Chinese adolescent resilience and mobile phone addiction. These results indicate the importance of mobile phone addiction and the importance of resilience for

adolescents. The findings may also help educators and medical personnel distinguish between predictive factors for adolescent mobile phone addiction; they could be used to design intervention to effectively treat and prevent mobile phone addiction in adolescents when dealing with future difficult and traumatic events.

List of abbreviations

DASS: depression, anxiety, and stress; MPAI: mobile phone addiction index; RSCA: the Resilience Scale for Chinese Adolescents ; SCSQ: the Simplified coping style questionnaire.

Acknowledgements

Not applicable.

References

1. China Internet Network Information Center. https://www.cnnic.net.cn/hlwfzyj/hlwxzbg/hlwtjbg/202109/P020210915523670981527.pdf

2. Patton GC, Sawyer SM, Santelli JS, et al. Our future: a Lancet commission on adolescent health and wellbeing.Lancet.2016;387:2423-2478.

3. Ozamiz-Etxebarria, N., Dosil-Santamaria, M., Picaza-Gorrochategui, M., & Idoiaga-Mondragon, N. Stress, anxiety, and depression levels in the initial stage of the COVID-19 outbreak in a population sample in the northern Spain. Cadernos de Saude Publica.2020;36:4,e00054020. https://doi. org/10.1590/0102-311X00054020

4.Guang-Li Lu,Yue-Ming Ding,Yi-Ming Zhang, Hai-Tao Huang, Yi-Pei Liang, Chao-Ran Chen. The correlation between mobile phone addiction and coping style among Chinese adolescents: a meta-analysis. Child and Adolescent Psychiatry and Mental Health.2021;15:60 . https://doi.org/10.1186/s13034-021-00413-2

5.The Tenth Press Conference of Henan Province Flood Control and Disaster Relief. Available online: http://www.henan.gov.cn/2021/08-02/2194036.html (accessed on 27 August 2021).

6. Enoka Randeniya. Flooding disaster: The effect on the adolescents at Angoda RahulaCollege, Sri Lanka. Procedia Engineering.2018;723–728.

7. Makwana N. Disaster and its impact on mental health: A narrative review. J Family Med Prim Care .2019;8:3090-5. DOI:10.4103/jfmpc.jfmpc_893_19.

8. Satadeepa Som, Sunil Kumar Giriyappa Patil. Gender differences in coping behaviour among adolescents.International Journal of Psychology.2019;12,2: 39-42. http://www.medpulse.in

9. Breik, Wisam,Zaza, Haidar.Coping strategies adopted by adolescents: A comparative study in relation to gifted status, gender, and family size.Gifted Education International.2019;35,1:3-19. DOI:10.1177/0261429418824118.

10. Davis SJ, Spillman S. Reasons for drug abstention: a study of drug use and resilience. J Psychoactive Drugs .2011;43:14-19.

11. Carli V, Durkee T, Wasserman D, Hadlaczky G, Despalins R, Kramarz E, et al. The association between pathological internet use and comorbid psychopathology: a systematic review. Psychopathology .2013;46:1-13.

12. Lazarus, R. S., and Folkman, S. Stress, Appraisal, and Coping. New York,NY: Springer1984.

13. Chapman, Paula L.; Mullis, Ronald L. Adolescent Coping Strategies and Self-Esteem. Child Study Journal.1999;29,1: 69-77.

14. Orgilés M, Morales A, Delvecchio E,et al. Coping Behaviors and Psychological Disturbances in Youth Affected by the COVID-19 Health Crisis. Front.Psychol. 2021;12:565-657.

15.Fang Liu, Zhonghao Zhang, Liang Chen.Mediating effect of neuroticism and negative coping style in relation to childhood psychological maltreatment and smartphone addiction among college students in China.Child Abuse & Neglect.2020;106. https://doi.org/10.1016/j.chiabu.2020.104531.

16.de Terte, I., & Stephens, C. Psychological resilience of workers in high- risk occupations (Editorial). Stress and Health: Journal of the International Society for the Investigation of Stress.2014;30,5: 353–355. https://doi.org/10.1002/smi.2627

17. Schriber, R. A. & Guyer, A. E. Adolescent neurobiological susceptibility to social context. Dev. Cogn. Neurosci.2016;19:1–18.

18. Southwick, S. M., Bonanno, G. A., Masten, A. S., Panter-Brick, C. & Yehuda, R. Resilience definitions, theory, and challenges: interdisciplinary perspectives.European Journal of Psychotraumatology .2014;5:25338.

http://dx.doi.org/10.3402/ejpt.v5.25338

19. Southwick, S. M. & Charney, D. S. The science of resilience: implications for the prevention and treatment of depression. Science .2012;338: 79–82.

20. Hu, T., Zhang, D. & Wang, J. A meta-analysis of the trait resilience and mental health. Pers. Individ. Dif.2015;76: 18–27 .

21. Malhi, G.S., Das, P., Bell, E. et al. Modelling resilience in adolescence and adversity: a novel framework to inform research and practice. Transl Psychiatry.2019;9:316. https://doi.org/10.1038/s41398-019-0651-y

22.Li J, Chen Y-p, Zhang J, Lv M-m,Välimäki M, Li Y-f, Yang S-l, Tao Y-x,e B-y, Tan C-x and Zhang J-p.The Mediating Role of Resilience and Self-Esteem Between Life Events and Coping Styles Among Rural Left-Behind Adolescents in China: A Cross-Sectional Study.Front. Psychiatry .2020;11:560556.doi: 10.3389/fpsyt.2020.560556

23. Wu Y, Sang Z, Zhang X-C, Margraf J.The Relationship Between Resilience and Mental Health in Chinese College Students: A Longitudinal Cross-Lagged Analysis. Front. Psychol.2020;11:108.doi: 10.3389/fpsyg.2020.00108

24. Smida, M., Khoodoruth, M. A. S., Al-Nuaimi, S. K., et al. Coping strategies, optimism and resilience factors associated with mental health outcomes among medical residents exposed to coronavirus disease 2019 in Qatar. Brain and Behavior.2021;11:e2320. https://doi.org/10.1002/brb3.2320

25. Lovibond, P.F.; Lovibond, S.H. The structure of negative emotional states: Comparison of the Depression Anxiety Stress Scales (DASS) with the Beck Depression and Anxiety Inventories. *Behav. Res. Ther*.1995;*33*:335–343.

26. Louis Leung. Linking psychological attributes to addiction and improper use of the mobile phone among adolescents in Hong Kong. Journal of Children and Media.2008;2(2):93-113. DOI:10.1080/17482790802078565

27. Yueqin Hu,Yiqun Gan. Development and validity of Resilience Scale for Chinese Adolescent. Acta Psychologica Sinica.2008;40(8)：902-912.

胡月琴, 甘怡群. 青少年心理韧性量表的编制和效度验证(J). 心理学报, 2008, 40(8)：902-912.

28.Ya-Ning Xie. A preliminary study on the reliability and validity of the Simplified Coping Style questionnaire. Chinese Journal Clinical Psychology.1998;6(2):114-115.

解亚宁. 简易应对方式量表信度和效度的初步研究(J). 1998, 6(2):114-115.

29.Baron,R.M.,Kenny,D.A.. The moderator–mediator variable distinction in social psychological research: Conceptual, strategic, and statistical considerations. Journal of Personality and Social Psychology.1986;51(6):1173-1182.

30. Hu, L. T., and Bentler, P. M.. Cutoff criteria for fit indexes in covariance structure analysis: conventional criteria versus new alternatives. Struct. Equ. Model.1999; 6(1):1–55.

31.Kline RB. Principles and Practice of Structural Equation Modeling. 2nd ed. New York, NY: Guildford Press;2005.

32. Robertson, T.W.; Yan, Z.; Rapoza, K.A. Is Resilience a Protective Factor of Internet Addiction? Comput. Hum. Behav.2018;78:255–260. https://doi.org/10.1016/j.chb.2017.09.027

33.Kumpfe K.L. and Bluth B. Parent/child transactional processes predictive of resilience or vulnerability to "substance abuse disorders". Substance Use & Misuse.2004;39: 671–698.

34. Mark Griffiths. A 'components' model of addiction within a biopsychosocial framework. Journal of Substance Use.2005;10(4): 191–197.

35. Lee Y.K., Chang C.T., Lin Y. and Cheng Z.H. The dark side of smartphone usage: Psychological traits, compulsive behavior and technostress.

Computers in Human Behavior, 2014； 31, 373–383.

36. Hou X.L., Wang H.Z., Guo C., Gaskin J., Rost D.H. and Wang J.L.

Psychological resilience can help combat the effect of stress on problematic social networking site usage. Personality & Individual Differences,2017； 109, 61–66.

37. Alan R, Guzel HS. The investigation of the relationship between smartphone addiction, and problem-solving skills and ways of coping with stress. Dusunen Adam The Journal of Psychiatry and Neurological Sciences .2020;33:244-253.

38. Lu GL, Ding YM, Zhang YM, Huang HT, Liang YP, Chen CR. The correlation between mobile phone addiction and coping style among Chinese adolescents: a meta-analysis. Child Adolesc Psychiatry Ment Health.2021;15(1):60. doi: 10.1186/s13034-021-00413-2.

39.Ozen S, Topcu M. The relationship of smartphone addiction with depression, obsession-compulsion, impulsivity, alexithymia among medical faculty students. Journal of Dependence .2017;18:16-24.

40.Elhai JD, Tiamiyu MF, Weeks JW, Levine JC, Picard KJ, Hall BJ. Depression and emotion regulation predict objective smartphone use measured over one week. Pers Individ Dif .2018;133:21-28.

41. Pera A . The Psychology of Addictive Smartphone Behavior in Young Adults: Problematic Use, Social Anxiety, and Depressive Stress. Front. Psychiatry. 2020;11:573473. doi: 10.3389/fpsyt.2020.573473

42. Sonali Tanmay Choksi; Nipa Patel. A Study to Find Out the Correlation of Mobile Phone Addiction with Anxiety, Depression, Stress and Sleep Quality in the College Students of Surat City. Int J Cur Res Rev .2021;13(8):137-142. DOI: http://dx.doi.org/10.31782/IJCRR.2021.13812.

43. Al. Qudah, M.F.; Albursan,I.S.; Hammad, H.I.; Alzoubi, A.M.;Bakhiet, S.F.; Almanie, A.M.; Alenizi,S.S.; Aljomaa, S.S.; Al-Khadher, M.M.Anxiety about COVID-19 Infection,and Its Relation to Smartphone Addiction and Demographic Variables in Middle Eastern Countries. Int. J. Environ. Res. Public Health.2021; 18: 11016. https://doi.org/10.3390/ijerph182111016.

44. Ahn, S. Y., Kim, Y. J. The influence of smart phone use and stress on quality of sleep among nursing students. Ind. J. Sci. Technol.2015;8:1–6. doi:10.17485/ijst%2F2015%2Fv8i35%2F85943

45. Einar B. Internet Addiction, Psychological Distress, and Coping Responses Among Adolescents and Adults. CYBERPSYCHOLOGY, BEHAVIOR, AND SOCIAL NETWORKING.2017;20(5):296-304. DOI: 10.1089/cyber.2016.0669

46. Stahl GK, Caligiuri P. The effectiveness of expatriate coping strategies: the moderating role of cultural distance, position level, and time on the international assignment. J Appl Psychol.2005;90:603–15. doi: 10.1037/0021-9010.90.4.603

47. Duan L, He J, Li M, Dai J, Zhou Y, Lai F and Zhu G . Based on a Decision Tree Model for Exploring the Risk Factors of Smartphone Addiction Among Children and Adolescents in China During the COVID-19 Pandemic. Front. Psychiatry. 2021;12:652356. doi: 10.3389/fpsyt.2021.652356

Supporting information

S1 Fig.

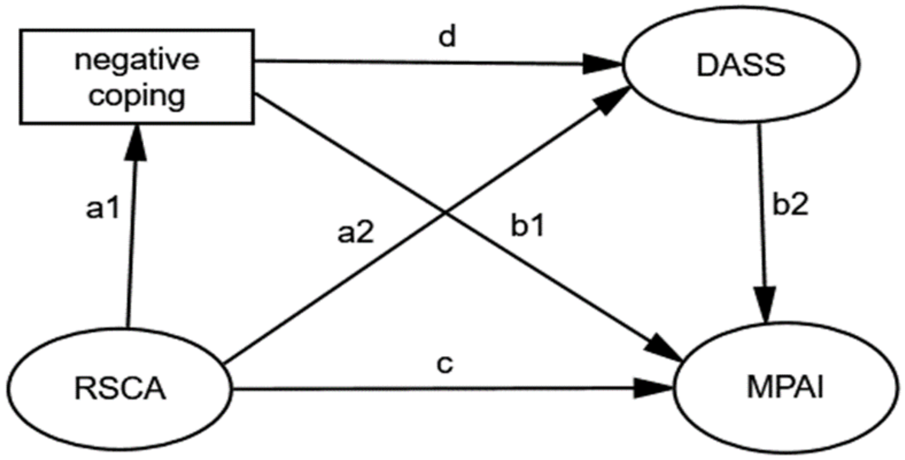

Figure 1 Hypothesized model

Notes: mobile phone addiction index (MPAI); depression, anxiety, and stress scale with

21 items (DASS-21); Resilience Scale for Chinese Adolescents (RSCA); Simplified coping style questionnaire (SCSQ).

S2 Fig.

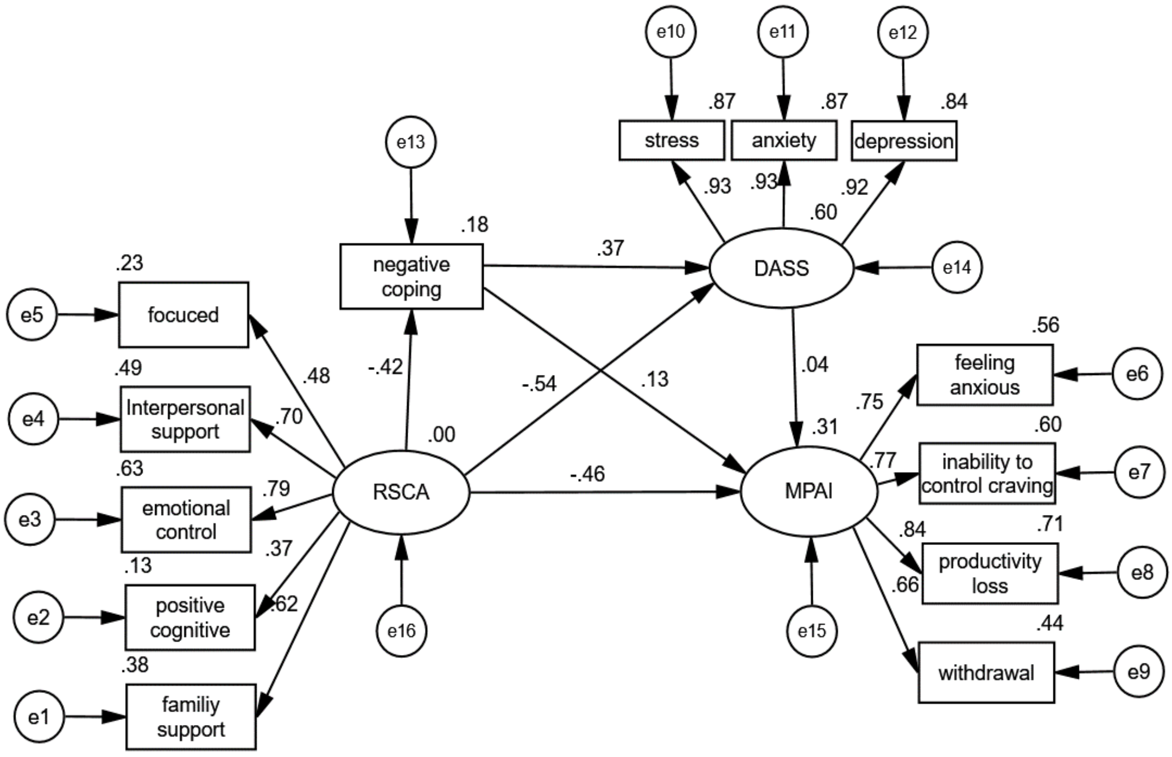

Figure 2 The standardized path coefficients in model testing

S1 Table.

Table 1: Demographic profiles and descriptive statistics of the participants.

**Gender**	Frequency	Percentage
boy	979	43.2
girl	1289	56.8
**One-child**		
yes	169	7.5
no	2099	92.5
**Birth order**		
1st	1048	46.2
2nd	1041	45.9
3rd	179	7.9
**Nationality**		
Han	2259	99.6
Hui	8	0.4
Miao	1	0.0
**Grade**		
middle school 7th	174	7.7
middle school 8th	634	28.0
middle school 9th	436	19.2
high school 1st	34	1.5
high school 2nd	471	20.8
high school 3rd	519	22.9
Total	2268	100.0

S2 Table.

	M	Std.Error of Mean	Frequency	Percentage
**Age**	14.90	2.58		
**MPAI Total**	39.57	13.82		
feeling anxious&lost	7.96	4.02		
inability to control craving	16.65	5.92		
productivity loss	7.64	3.28		
withdrawal	7.32	3.49		
**DASS-21 Total**	5.19	4.57		
**Stress**	7.40	5.07		
normal			2054.00	90.56
mild			124.00	5.47
moderate			76.00	3.35
severe			14.00	0.62
extremely severe			0.00	0.00
**Anxiety**	6.31	4.94		
normal			1583.00	69.80
mild			218.00	9.61
moderate			304.00	13.40
severe			103.00	4.54
extremely severe			60.00	2.65
**Depression**	6.58	5.09		
normal			1773.00	78.18
mild			258.00	11.38
moderate			179.00	7.89
severe			48.00	2.12
extremely severe			10.00	0.44
**SCSQ Total**	56.39	17.91		
positive coping	22.45	9.18		
negative coping	11.93	5.64		
**RSCA Total**	88.89	18.50		
focuced	16.24	4.88		
Interpersonal support	18.51	5.90		
emotional control	19.63	5.76		

Positive cognitive	14.30	3.78
familiy support	16.77	4.50

Table 2. Basic characteristics and measure scores.

S3 Table.

Table3: Correlation analysis of study variables

	1	2	3	4
**1.RSCA Total**	1			
**2.Negative coping**	-.317**	1		
**3.DASS-21 Total**	-.593**	.603**	1	
**4.MPAI Total**	-.405**	.322**	.448**	1

Note: ***P* < 0.01

S4 Table.

Table 4. Results of the structural model: tests of hypothesized associations between constructs.

			Estimate	S.E.	t-value	P
**negative coping**	**<---**	**RSCA**	-1.025	0.078	-13.216	***
**DASS**	**<---**	**negative coping**	0.292	0.017	16.871	***
**DASS**	**<---**	**RSCA**	-1.152	0.061	-18.992	***
**MPAI**	**<---**	**RSCA**	-0.836	0.07	-11.919	***
**MPAI**	**<---**	**negative coping**	0.09	0.017	5.355	***
**MPAI**	**<---**	**DASS**	-0.096	0.031	-3.092	0.002

Note: ***P < 0.01

S5 Table.

Table 5. Bootstrap truncated regression results.

		Product of coefficients		Bootstrapping				
				BC 95%CI		Percentile 95%CI		
Relationships	**point estimate**	***SE***	***Z***	**Lower**	**Upper**	**Lower**	**Upper**	***P***
			Indirect Effects					
**RSCA → negative coping → MPAI**	-0.092	0.018	-5.111	-0.125	-0.061	-0.125	-0.061	0
**RSCA → DASS → MPAI**	0.111	0.043	2.581	0.043	0.188	0.045	0.186	0.002
**RSCA → negative coping → DASS→ MPAI**	0.029	0.011	2.636	0.012	0.048	0.012	0.048	0.002
**Total**	-0.789	0.052	-15.173	-0.889	-0.698	-0.89	-0.699	0

Click here to access/download;Figure;Figure 1 Hypothesized model.pdf

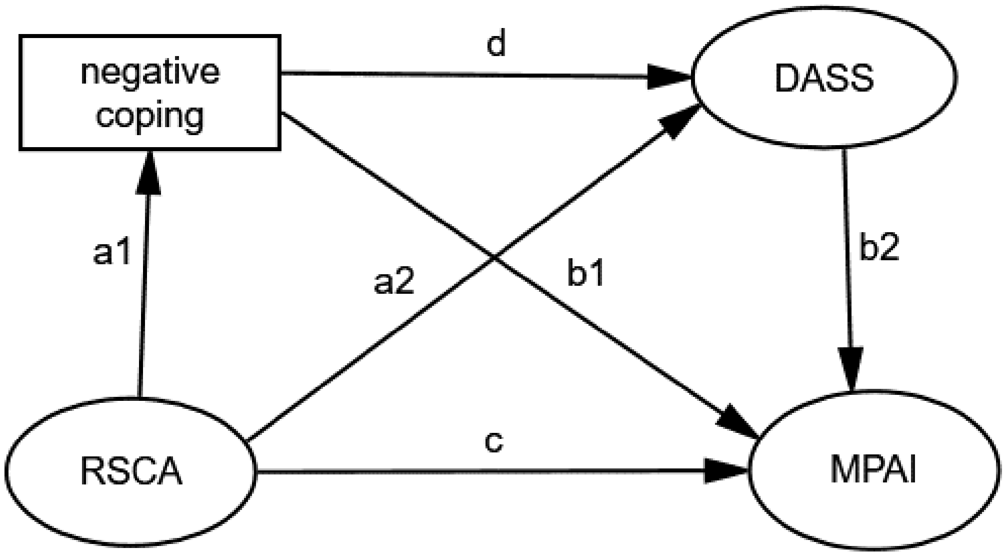

Figure 1 Hypothesized model

Notes: mobile phone addiction index (MPAI); depression, anxiety, and stress scale with 21 items (DASS-21); Resilience Scale for Chinese Adolescents (RSCA); Simplified coping style questionnaire (SCSQ).

Click here to
access/download;Figure;Figure_2.The_standardized_path_coefficients_in_model_tes

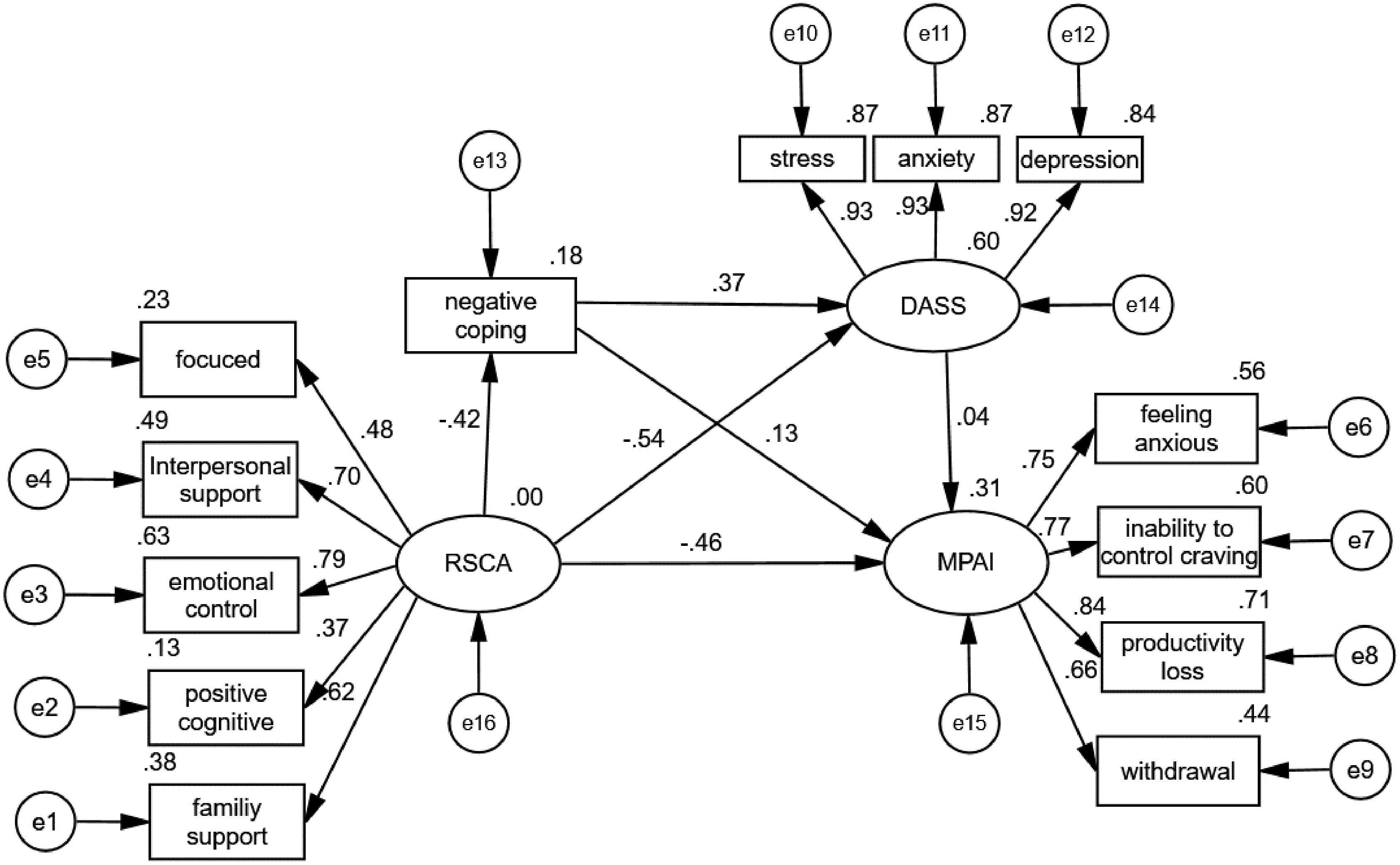

Figure 2 The standardized path coefficients in model testing

Click here to access/download
Supporting Information
tables.docx
