# Supplementary material for: Adolescent resilience and mobile phone addiction in Henan Province of China: Impacts of chain mediating, coping style
Source: PLoS One. 2022 Dec 27;17(12):e0278182. doi: 10.1371/journal.pone.0278182 (PMC9794036; doi:10.1371/journal.pone.0278182)
Supplement: Attachment — Submitted filename: PONE-D-22-09528_R1_reviewer.tcsw02.pdf [file pone.0278182.s004.pdf]

PLOS ONE

Adolescent Resilience and Mobile Phone Addiction in Henan province of China: Impacts of Chain Mediating, Coping Style

--Manuscript Draft--

**Manuscript Number:**	PONE-D-22-09528R1
**Article Type:**	Research Article
**Full Title:**	Adolescent Resilience and Mobile Phone Addiction in Henan province of China: Impacts of Chain Mediating, Coping Style
**Short Title:**	The relationship between psychological resilience and mobile phone addiction in adolescents
**Corresponding Author:**	Anna Ma<br>Xinxiang Medical University<br>Xinxiang, CHINA
**Keywords:**	Keywords: Adolescent resilience; coping style; Mobile phone addiction; China; dass-21; Chain mediating
**Abstract:**	Abstract<br>Background: As mobile phone use grows, so it brings benefits and risks. As an important part of adolescents healthy growth, resilience plays an indispensable role . Thus, it is important to identify when mobile phone use of an adolescent becomes an addiction. This study proposed to explore the effects of adolescent resilience on mobile phone addiction, and tested the mediating role of coping style and depression, anxiety, and stress (DASS) on phone addiction among 2,268 adolescents in the Henan province,China.<br>Methods: The adolescents were surveyed via an online questionnaire,a mobile phone addiction index (MPAI), a depression, anxiety, and stress scale with 21 items (DASS-21), the Resilience Scale for Chinese Adolescents (RSCA), and the Simplified coping style questionnaire (SCSQ), and we used structural equation modeling to examine the correlations and moderation effects. All data analyses were performed using SPSS 26.0 and Amos 23.0.<br>Results: The results show that adolescences resilience were negatively related to negative coping ,DASS, and mobile phone addiction;both coping style and DASS could mediate the relationship between adolescent resilience and mobile phone addiction among Chinese adolescents. The relationship between adolescent resilience and mobile phone addiction in Chinese adolescents was mediated by the chain of coping styles and DASS.<br>Conclusions: There is a negative relationship which exists between resilience and mobile phone addiction in this population. In addition, stress, anxiety, depression, and coping style significantly influence the risk of adolescent mobile phone addiction and play an intermediary role in Chinese adolescent resilience and mobile phone addiction.
**Order of Authors:**	Anna Ma<br>Hongjuan Chang<br>Yan Yang<br>Shuangxi Guo<br>Xue Li<br>Shenhua Zhang
**Response to Reviewers:**	Reviewer #1: This article may be corrected as follows :<br>1. In abstract to be improved to describe succinctly related to Introduction, Method, Result and Discussion, on a good abstract will also be seen what becomes novelty of the article.<br>2. The introduction must be explained two main aspects; there are aspects of the theory that is backgrounded and problems found in the field to look research gap from this article. Then at the end of the introduction will be able to see what is new from this study.

Powered by Editorial Manager® and ProduXion Manager® from Aries Systems Corporation

	3. The methodology may need to be explained in more detail related to the research subject so that it becomes clear who the subject is.<br>4. The result and discussion should give the table and figure of results more in-depth, and you have not done.<br>5. Conclusions must undoubtedly be able to answer from the purpose of this study.<br>Response<br>Thank you for giving this suggestion.We revise the abstract section and introduction,and detail explain the subject,amend the result,discussion and conclusion.<br><br>Reviewer #2: Link between resilience and mobile phone addiction not made clear.<br>Resilience not well defined, how is it being applied and to what purpose?<br>What definition of mobile phone addiction? When does use become addiction? Why is this important?<br>AS well as consent from parents – did young people themselves get a chance to consent or at least give assent?<br>Last sentence page 3 seems out of place – reads like findings.<br>Greater focus on the impact of and recovery from crisis such as covid, on mobile phone use, resilience and mental and social, wellbeing would make a clearer rationale for the study.<br>The impact of covid could have been considered beyond the stress and anxiety it caused. It is natural to expect through lockdowns and social restrictions that adolescents would have become more reliant on mobile phones. The mention of covid here feels somewhat tokenistic and not very well thought through.<br>Greater explanation of methodology and methods would make it clearer why and how this research was done the way it was.<br>Discussion of the findings limits the usefulness of the research. It could be made much ore clear what the links are between main components of the research and the future implications of this for Chinese adolescents.<br>Some written English and referencing issues to be corrected.<br>Response<br>Thank you for giving this comments. We have remend the introduction of this article to emphasize definition of Resilience and mobile phone addiction,and explains the application of resilience in many studies.We explain the harm of mobile phone addiction to adolescents.The study complied with ethical requirements and was carried out on the basis of the consent of the parents or guardians of minors and adolescent themselves.We also revise the methods ,result ,discussion and conclusion section, the written English and referencing are corrected in the article.
**Additional Information:**	
**Question**	**Response**
**Financial Disclosure**<br><br>Enter a financial disclosure statement that describes the sources of funding for the work included in this submission. Review the submission guidelines for detailed requirements. View published research articles from *PLOS ONE* for specific examples.<br><br>This statement is required for submission and **will appear in the published article** if the submission is accepted. Please make sure it is accurate.	This work was supported by the National Natural Science Foundation of China (grant number: 81803252).This funder had no role in the design of the study and collection, analysis, and interpretation of data and in preparation and writing the manuscript.

**Unfunded studies**<br>Enter: *The author(s) received no specific funding for this work.*<br><br>**Funded studies**<br>Enter a statement with the following details:<br>• Initials of the authors who received each award<br>• Grant numbers awarded to each author<br>• The full name of each funder<br>• URL of each funder website<br>• Did the sponsors or funders play any role in the study design, data collection and analysis, decision to publish, or preparation of the manuscript?<br>• **NO** - Include this sentence at the end of your statement: *The funders had no role in study design, data collection and analysis, decision to publish, or preparation of the manuscript.*<br>• **YES** - Specify the role(s) played.<br><br>* typeset	
**Competing Interests**<br><br>Use the instructions below to enter a competing interest statement for this submission. On behalf of all authors, disclose any competing interests that could be perceived to bias this work—acknowledging all financial support and any other relevant financial or non-financial competing interests.<br><br>This statement is required for submission and **will appear in the published article** if the submission is accepted. Please make sure it is accurate and that any funding sources listed in your Funding Information later in the submission form are also declared in your Financial Disclosure statement.<br><br>View published research articles from *PLOS ONE* for specific examples.	The authors have declared that no competing interests exist.

**NO authors have competing interests**<br><br>Enter: *The authors have declared that no competing interests exist.*<br><br>**Authors with competing interests**<br><br>Enter competing interest details beginning with this statement:<br><br>*I have read the journal's policy and the authors of this manuscript have the following competing interests: [insert competing interests here]*<br><br>* typeset	
**Ethics Statement**<br><br>Enter an ethics statement for this submission. This statement is required if the study involved:<br><br>• Human participants<br>• Human specimens or tissue<br>• Vertebrate animals or cephalopods<br>• Vertebrate embryos or tissues<br>• Field research<br><br>Write "N/A" if the submission does not require an ethics statement.<br><br>General guidance is provided below. Consult the submission guidelines for detailed instructions. **Make sure that all information entered here is included in the Methods section of the manuscript.**	The participants were under 18 years old, and we provided written informed consent to their parents (or legal guardians)before taking part in the study .The procedures performed in the study were approved by the Ethics Committee ofXinxiang Medical University(number:XYLL-2018015),as are all studies involving human participants. The data from the participants were de-identified.

Powered by Editorial Manager® and ProduXion Manager® from Aries Systems Corporation

**Format for specific study types**<br><br>**Human Subject Research (involving human participants and/or tissue)**<br>• Give the name of the institutional review board or ethics committee that approved the study<br>• Include the approval number and/or a statement indicating approval of this research<br>• Indicate the form of consent obtained (written/oral) or the reason that consent was not obtained (e.g. the data were analyzed anonymously)<br><br>**Animal Research (involving vertebrate animals, embryos or tissues)**<br>• Provide the name of the Institutional Animal Care and Use Committee (IACUC) or other relevant ethics board that reviewed the study protocol, and indicate whether they approved this research or granted a formal waiver of ethical approval<br>• Include an approval number if one was obtained<br>• If the study involved *non-human primates*, add *additional details* about animal welfare and steps taken to ameliorate suffering<br>• If anesthesia, euthanasia, or any kind of animal sacrifice is part of the study, include briefly which substances and/or methods were applied<br><br>**Field Research**<br><br>Include the following details if this study involves the collection of plant, animal, or other materials from a natural setting:<br>• Field permit number<br>• Name of the institution or relevant body that granted permission	
**Data Availability**<br><br>Authors are required to make all data underlying the findings described fully available, without restriction, and from the time of publication. PLOS allows rare exceptions to address legal and ethical concerns. See the PLOS Data Policy and FAQ for detailed information.	No - some restrictions will apply

A Data Availability Statement describing where the data can be found is required at submission. Your answers to this question constitute the Data Availability Statement and **will be published in the article**, if accepted.<br><br>**Important:** Stating 'data available on request from the author' is not sufficient. If your data are only available upon request, select 'No' for the first question and explain your exceptional situation in the text box.<br><br>Do the authors confirm that all data underlying the findings described in their manuscript are fully available without restriction?	
**Describe where the data may be found in full sentences. If you are copying our sample text, replace any instances of XXX with the appropriate details.**<br><br>• If the data are **held or will be held in a public repository**, include URLs, accession numbers or DOIs. If this information will only be available after acceptance, indicate this by ticking the box below. For example: *All XXX files are available from the XXX database (accession number(s) XXX, XXX.)*.<br>• If the data are all contained **within the manuscript and/or Supporting Information files**, enter the following: *All relevant data are within the manuscript and its Supporting Information files.*<br>• If neither of these applies but you are able to provide **details of access elsewhere**, with or without limitations, please do so. For example:<br><br>*Data cannot be shared publicly because of [XXX]. Data are available from the XXX Institutional Data Access / Ethics Committee (contact via XXX) for researchers who meet the criteria for access to confidential data.*<br><br>*The data underlying the results presented in the study are available from (include the name of the third party*	Due to the potential for indirect adolescents and their parents recognition of this adolescent subgroup, the datasets generated during the current study hide sensitive and identity information which are included in the article/supplementary material.

and contact information or URL).<br>• This text is appropriate if the data are owned by a third party and authors do not have permission to share the data.<br><br>* typeset	
Additional data availability information:	Tick here if your circumstances are not covered by the questions above and you need the journal's help to make your data available.

Powered by Editorial Manager® and ProduXion Manager® from Aries Systems Corporation

Manuscript

Click here to access/download;Manuscript;renamed_a8c74.docx

Adolescent Resilience and Mobile Phone Addiction in Henan Province of China: Impacts of Chain Mediating, Coping Style

Anna Ma[1 #a #b], Yan Yang[1], Shuangxi Guo[2], Xue Li[1], Shenhua Zhang[3], Hong Juan Chang[1*]

[1 #a] Xinxiang Medical University, School of Nursing, Xinxiang, Henan province,China

[#b] St. Paul University, School of Nursing, Manila, Philippines

[2]The First Affiliated Hospital of Xinxiang Medical University, Department of Neurology, Xinxiang, Henan province, China

[3] Weihui Senior Middle School, Xinxiang, China

*** Correspondence:**

Hong Juan Chang

E-mail: changhj0812@126.com

Conflict of Interest/Disclosure Statement

Competing interests

The authors declare that they have no competing interests and all methods were carried out in accordance with relevant guidelines and regulations. The authors alone are responsible for the content and writing of the article.

Author Contributions

Hong Juan Chang designed the study, Xue Li and Shenhua Zhang collected the data, Anna Ma analyzed the data and wrote the manuscript. Yan Yang and Shuangxi Guo revised the manuscript. All authors contributed to the article and approved the submitted version.

Acknowledgements

Not applicable.

Abstract

Background: As mobile phone use grows, so it brings benefits and risks. As an important part of adolescents healthy growth, resilience plays an indispensable role . Thus, it is important to identify when mobile phone use of an adolescent becomes an addiction. This study proposed to explore the effects of adolescent resilience on mobile phone addiction, and tested the mediating role of coping style and depression, anxiety, and stress (DASS) on phone addiction among 2,268 adolescents in the Henan province,China.

Methods: The adolescents were surveyed via an online questionnaire,a mobile phone addiction index (MPAI), a depression, anxiety, and stress scale with 21 items (DASS-21), the Resilience Scale for Chinese Adolescents (RSCA), and the Simplified coping style questionnaire (SCSQ), and we used structural equation modeling to examine the correlations and moderation effects. All data analyses were performed using SPSS 26.0 and Amos 23.0.

Results: The results show that adolescences resilience were negatively related to negative coping ,DASS, and mobile phone addiction;both coping style and DASS could mediate the relationship between adolescent resilience and mobile phone addiction among Chinese adolescents. The relationship between adolescent resilience and mobile phone addiction in Chinese adolescents was mediated by the chain of coping styles and DASS.

Conclusions: There is a negative relationship which exists between resilience and mobile phone addiction in this population. In addition, stress, anxiety, depression, and

coping style significantly influence the risk of adolescent mobile phone addiction and play an intermediary role in Chinese adolescent resilience and mobile phone addiction.
Keywords: Adolescent resilience; coping style; mobile phone addiction; China; DASS-21; chain mediating

Introduction

With the development of information technology, the functions of a mobile phone are becoming more and more powerful; it is now considered as an important communication tool and social accessory.However,increased dependency brings a downside, namely,smartphone phone addiction.Smartphone addiction consists of four main components: obsessive phone use and compulsive behaviors; tolerance of longer and more intense of use; withdrawal or suffering without the phone; and functional impairment [1].

Due to the COVID-19 outbreak, mobile phones have become increasingly important for online teaching and learning in China. According to the 48th China statistical report on internet development, by June 2021, there were 1.07 billion mobile phone users in China, accounting for 99.7% of the total number of internet users. Furthermore, internet users spend an average of 26.9 hours online per week, and the number of internet users between the ages of 6 and 19 reached 158 million, accounting for 15.7% of the total [2]. As part of normal adolescent psychological development, this age group develops susceptibility to peer influences and tends to have low-risk perception—factors that can result in increased risk-taking behavior and poor self-regulation [3]. Stressful life events

have been identified as a significant risk factor in the emotional state of adolescents. The spread of COVID-19 has been one of these stressful life events as it led to substantial social and economic changes as a result of many governments introducing quarantine policies to prevent the virus from spreading and thus assure the safety of the population. Psychopathologists have focused on the psychological impact of COVID-19 and its variations in the adolescent population and they have identified moderate to severe levels of stress, anxiety, and depression in this population [4]. Guang-Li found that a moderate positive correlation between negative coping style and adolescents' mobile phone addiction[5].

In the middle of July 2021, Henan province suffered from unusually heavy rainfall (continuous rainfall of 958 mm[6]), causing severe flooding. The flood, named the "7.20 Henan rainstorm," overwhelmed dams and burst riverbanks in a short time, causing severe traffic paralysis, power failure, loss of crops, and upending tens of millions of lives. Randeniya reported that after this natural disaster, adolescents of Sri Lanka were mostly affected by sleeping difficulties [7]. Makwana indicated that the psychological effects of the disaster were fiercer among children, women, and the dependent elderly population [8].

The general strain theory argues that adolescents can be pressured into delinquency by a negative experience,this negative effect creates pressure for corrective action and may lead adolescents to escape from the source of their adversity, or manage the negative effect through the use of illicit drugs[9]. The general strain theory was originally used for adolescents' criminal behavior, but now many researchers use it for explaining addictive behaviors [10-

12].Recent studies have argued that stress and disaster can cause mobile phone addiction through various pathways[13-15].Disaster can lead to severe stress,uncontrollable stress,and substance dependency of the individual[16].According to many studies, natural disasters and COVID-19 as stressors can lead people to depression, anxiety, substance abuse,and addictive behavior[17,18].Some experts also believe that depression, anxiety, and pressure may lead to internet addiction[19].Research has also shown that exposure to disaster is positively associated with substance abuse and is negatively related to children's psychological resilience[20]. Resilience is commonly described as the ability to bounce back or overcome some form of adverse event or situation and thus experience positive outcomes [21].Resilience is an important developmental stage during adolescence, as it is a transitional period characterized by significant neurobiological and psychosocial changes in the context of amplifying environmental demands and increasing sensitivity to social contexts [22].One study has found that there is a negative relation between high psychological resilience and depression[23].Resilience is also a protective factor of excessive smartphone use[24].Many scholars believe that adolescent resilience is also related to substance abuse, such as smoking and excessive drinking (alcoholism) [25]. However, studies on the relationship between mental resilience and adolescents' coping styles, mental health, and mobile phone addiction are rare.

In view of this, adolescents confined to their homes due to the floods in Henan may have overused their mobile phones and the internet. Many studies have shown that mobile phone addiction has had a negative impact on physical and mental health as well

as social adaption,academic achievement, and sleep quality[26-28].Thus, we hypothesized that there is a correlation between resilience and mobile phone addiction among adolescents and that coping style and mental health play a mediating role in that relationship.

“Coping” means constantly changing cognitive and behavioral efforts to manage specific external and/or internal demands that are appraised as taxing or exceeding a person’s resources [29]. Chapman thought that adolescents with lower self-esteem engage in coping strategies of ventilating feelings, avoiding problems, and relaxing, while adolescents with higher self-esteem were more likely to engage in coping styles that directly address solving the problem[30]. During the COVID-19 pandemic, children who used positive strategies to cope with the situation suffered less emotionally and behaviorally [31]. Fang Liu found that a negative coping style mediated the relationship between smartphone addiction and childhood psychological maltreatment[32]. Psychological resilience is the ability to cope with a crisis or to quickly return to a pre-crisis status, either mentally or emotionally [33].

Resilience negatively predicted a negative coping style and positively predicted a positive coping style. Life events not only directly influenced a negative coping style and positive coping style but also indirectly influenced coping styles by affecting resilience[34]. The mutually enhancing relationship between resilience and positive mental health, and vice versa, a mutually reducing relationship between resilience and mental illness, and presented the significant influence of mental health level on resilience[35]. Malek showed that the coping style of avoidance could aggravate

depressive, anxiety, and stress symptoms of depression, anxiety, and stress in participants during the COVID- 19 pandemic. Keeping optimism, resilience, and approach coping styles decreased the mental health burden of the pandemic on participants [36].

The present study

Based on the literature review above, the relationship between adolescent resilience and mobile phone addiction deserves attention.The present study constructed a chain mediation model to examine the mediating role of negative coping, stress, anxiety, and depression in the relationship between adolescent resilience and mobile phone addiction among Chinese adolescents. Furthermore, we proposed a model to test the associations among Chinese adolescent resilience, coping style, mental health, and mobile phone addiction, to further clarify mobile phone addiction related to resilience (Fig 1).

Materials and Methods

Participants

The convenience sampling method was employed to select adolescents from middle school and high school in Henan province of China to complete the questionnaires online from July 1 to August30, 2021. In total, 2,268 valid questionnaires were obtained, with an effective rate of 97.28%. Among them, the mean age was 14.90 years (*SD* = 2.58, range = 12–21 years), participants including 979 boys(43.20%)and 1,289 girls (56.80%). They completed a survey that included demographic variables, a

mobile phone addiction index (MPAI), a depression, anxiety, and stress scale with 21 items (DASS-21), the Resilience Scale for Chinese Adolescents (RSCA), and the Simplified Coping Style questionnaire (SCSQ).The link to the questionnaires was sent out to potential participants. All questions were completed by respondents clicking on the appropriate option.In the case of a missing answer, the server would remind them to give the missing answer. All participants filled in the questionnaire anonymously, without any payment.Their privacy was protected.

Ethics approval and consent to participate

The participants were under 18 years old, and we provided written informed consent to their parents (or legal guardians)before taking part in the study . The procedures performed in the study were approved by the Ethics Committee of Xinxiang Medical University(number:XYLL-2018015),as are all studies involving human participants. The data from the participants were de-identified.

Measurement of Structures

DASS-21

The DASS-21was used to evaluate negative emotional states of depression, anxiety, and stress[37] and refer to the previous week. In the DASS-21,each item is classified into four Likert responses from 0 to 3, where 0 = “nothing” and 3 = “Most of the time.” This self-report instrument includes three subscales: 1) the stress subscale, which measures tension, agitation, difficulty relaxing, and negative affection; 2)the anxiety subscale, which assesses autonomic arousal, skeletal musculature effects,

situational anxiety, and subjective experience of anxiety arousal; and 3) the depression subscale, which measures hopelessness, dysphoria, lack of interest, self-deprecation, and inertia. The reliability coefficients of depression, anxiety and stress were 0.82, 0.82 and 0.79, respectively. The Cronbach's alpha of the total scale was 0.89.

MPAI

The MPAI was designed by Louis Leung to identify addiction symptoms associated with mobile phone use among adolescents in Hong Kong[38]. The scale includes 17 items answered on a five-point Likert scale of 1 to 5 (1 = not at all; 2 = rarely; 3 = occasionally; 4 = often; and 5 = always). The scale covers four dimensions: 1) "inability to control craving," which reflects the amount of time an adolescent spends on the mobile phone, thereby leading to complaints from family and friends about their compulsive mobile phone use and causing the adolescent's loss of sleep due to the excessive use; 2) "Anxiety and feeling lost" assesses preoccupation, feeling lost or anxious, and having difficulty switching off the mobile phone; 3)"Productivity loss" measures decreased productivity and diverted attention from pressing issues due to an adolescent's excessive use mobile phones; 4) "Withdrawal and escape" indicates that an adolescent uses their mobile phones to escape from isolation, loneliness, and feeling down. The Cronbach's alpha of scale was 0.90.

RSCA

The RSCA was developed by Yueqin Hu[39]according to the process model of the resilience concept and applied to Chinese adolescents. There are 27 items divided into two factors: “manpower” and “support”.The former includes three factors: goal focus, emotion control, and positive cognition while latter includes two factors: family support and interpersonal assistance. The reliability of the total scale was 0.85.

SCSQ

This SCSQ was designed by Ya-Ning Xie [40], composed of 20 items,Coping styles can be divided into two groups based on how people react to setbacks: “positive coping” and “negative coping." The reliability of the total scale was 0.90, while the positive coping and negative response subscales were 0.89 and 0.78, respectively.

Data Analysis

All data analyses were performed using SPSS 26.0 and Amos 23 (IBM Inc., Armonk, NY, USA). First, descriptive data were received using SPSS 26.0, and correlations variables were calculated using Pearson’s correlations. Second, according to Baron and Kenny[41], we analyzed the mediation effects using two measurement models to examine how well the indicators represented each latent variable. Second, we tested the hypothesized relationships among latent variables. Maximum likelihood (ML) estimation was used to test the two structural models in the AMOS 23.0 program. When TLI > 0.90, CFI > 0.90, and RMSEA < 0.06, the model fits well, according to

Hu and Bentler [42].We followed the stepwise method to structure the best-fitting model for the mediated effects and bootstrapping with 5,000 replications to measure the chain mediation model. All data analyses were two-tailed, with significance levels of $P < 0.01$ and $P < 0.05$.

Results

Descriptive Statistics

We included 2,268 participants, including 979 boys (43.20%) and 1,289 girls (56.80%), in the final analysis. The proportion of girls was slightly higher than that of boys (56.80% vs. 43.20%). The mean age was 14.90 years (SD = 2.58, range 12–21 years). There were 1244 (54.85%) participants from middle school, 1,024 (45.15%) from high school, 169 (7.50%) from one-child families, and 2,099 (92.50%) from multi-child families. The other results are shown in Table 1.

Univariate Analysis

The results for the 2,268 participants are displayed in Table 2. The category totals as total mean (*SD*) were as follows: MPAI, 39.57 (±13.82); DASS-21, 5.190 (±4.57); positive coping was 22.45 (±9.18); negative coping, 11.93 (±5.64); RSCA, 88.89 (±18.50).

Correlation Analysis of Major Study Variables

The variables correlated with the constructs in Table 3 were less than 0.85. The discriminant validity value (< 0.85) was met in the construct correlation[43]. These findings showed that valid and reliable constructs were used.We found that adolescents resilience were negatively related to negative coping ,DASS, and mobile phone addiction. Negative coping was positively related to both DASS and mobile phone addiction. The results of analyses also showed that adolescent resilience correlated with negative coping ($r = -.317$, $P< 0.01$) , DASS($r =-.593$,$P< 0.01$),and mobile phone addiction($r=-.405$,$P<0.01$);negative coping correlated with DASS($r=.603$,$P<0.01$),mobile phone addiction($r=.322$,$P<0.01$);DASS correlated with mobile phone addiction($r=.448$,$P<0.01$).Adolescents with poor resilience usually adopted a negative coping style and had higher anxiety, depression and mobile phone addiction—which confirms the hypothesis .

Structural Model Testing and Structural Relationship Between Constructs

The test results revealed the goodness of fit of the proposed structural model ($\chi2/df$ = 2.57, RMSEA = 0.054, GFI = 0.978, CFI = 0.984). The hypothesis relationships between the variates are demonstrated in Table 4 . Results of the test for path(negative coping <---RSCA) revealed a significant negative relationship between resilience and negative coping (β = -1.025, t = -13.216, $P < 0.01$). Results of the test for path(DASS <---negative coping) showed a significant positive relationship between DASS and negative coping (β = 0.292, t = 16.871,$P < 0.01$). Results of the test for path(DASS <---RSCA) revealed a significant negative relationship between DASS and resilience

($\beta = -1.152$, $t = -18.992$, $P < 0.01$). Results of the test for path(MPAI <---RSCA)showed a significant negative association between mobile phone addiction and resilience ($\beta = -0.836$, $t =-11.919$, $P < 0.01$). Results of path(MPAI <---negative coping)showed a significant relationship positive relationship between mobile phone addiction and negative coping ($\beta = 0.09$, $t = 5.355$, $P < 0.01$). Results of the test for path(MPAI <---DASS)revealed a significant negative relationship between mobile phone addiction and DASS ($\beta = -0.096$, $t = -3.092$, $P = 0.002$). Finally, the results confirmed the hypothesis.The variables were explained by their respective preceding construct (Fig 2)The indirect effects are presented in Table 5. Bootstrapping (the process was repeated 5,000 times)analyses showed that the indirect effects of adolescent resilience on mobile phone addiction through negative coping and stress, anxiety, and depression were significant and positive (standardized indirect effect 0.029,95%CI [0.012,0.048], $P < 0.01$), and the indirect effect of adolescent resilience on mobile phone addiction through stress, anxiety, depression was 0.111, 95% CI [0.045, 0.186], $P < 0.01$, excluding 0, and the mediating effect was significant. The indirect effect of adolescent resilience on mobile phone addiction through negative coping was -0.092, 95% CI [-0.125, -0.061], $P < 0.01$, excluding 0, and the mediating effect was significant.

Discussion

This study surveyed the ways by which adolescent psychological resilience, coping style and depression, anxiety, and stress affected mobile phone addiction among

Chinese adolescents.The main aim of the study was to establish if there was a relationship between resilience and mobile phone addiction in Chinese adolescents and if negative coping and DASS were a chain mediator of this relationship. The results showed that adolescent psychological resilience could directly and negatively affect mobile phone addiction in Chinese adolescents, in consequence,poor resilience may cause mobile phone addiction in Chinese adolescents,which is consistent with previous research findings .

According to the psychological resilience framework theory[44], psychological resilience is an important protective factor for problem behavior and personal mental health. Griffiths argued that addictions consist of several components, such as relapse, mood modification, tolerance, conflict, and withdrawal [45]. In the studies, psychological resilience was found to protect the addictive behaviors (internet problematic use)[46,47].Adolescent mobile phone addiction affect their life and study, and this study suggests that family, peer, teacher support, and exercise help to improve the depressive moods and make it more resilient to adversity and stress.

The experimental results reveal that adolescents psychological resilience is negatively correlated with negative coping style: negative coping style is negatively correlated with mobile phone addiction. It shows that adolescents with good psychological resilience are more likely to be adopt positive coping styles when facing pressure and frustration and are less likely to addicted to mobile phones.Francisco found that higher scores on perceived stress represented lower resilience and lower use of the

coping strategies of problem-focusing[48].Li et al. [49]also found that resilience could negatively predict the negative coping style and positively predict the positive coping style ,which is consistent with the results of this study.He et al. [50] found that adolescents adopt a negative coping style, which leads to mobile phone addiction. This study reveals that depression, anxiety, and stress (DASS)is negatively correlated with mobile phone addiction. Gao et al found that perceived stress was directly associated with mobile phone addiction[51].A large number of studies have shown that mobile phone addiction is positively associated with anxiety and depression,which in turn brings about severe adverse effects on mental health,such as anxiety ,depression and poor sleep quality[52-55].These studies suggest an interplay between anxiety, depression, and phone addiction.

These results show that negative coping styles and depression, anxiety, and stress (DASS) play intermediary roles, respectively, in adolescent resilience and mobile phone addiction in Chinese adolescents; thus, our hypothesis was verified. This finding is consistent with the results of previous studies.For example, Stanković found partial mediation between internet addiction and depression through stress and anxiety[56].

The Simplified Coping Style Questionnaire(SCSQ) results revealed that coping style had a maximal effect on adolescent mobile phone addiction[57]. Many studies have indicated a relationship between depression, anxiety, and loneliness with smartphone usage [58,59]. Depression and social anxiety are risk determinants for greater

problematic smartphone use [60]. Stress, anxiety, and depression were significantly positively correlated with smartphone addiction [61]. Researcher have found that a significant positive relationship between anxiety about COVID-19 infection and daily smartphone use hours; the largest predictor of smartphone addiction was anxiety about COVID-19 infection [62].

Negative coping style and DASS played a continuous intermediary role in the impact of adolescent resilience on mobile phone addiction among Chinese adolescents. Smartphone users who experience depressive symptoms may similarly use their mobile devices as a coping strategy to alleviate these unpleasant symptoms [63]. Coping and affective disorders appear to play a key role in international addiction among adolescents[64]. COVID-19 and floods as stressors can cause psychological stress responses in adolescents, and differences in coping styles can cause differences in behaviors in adolescents. Coping style is a significant factor leading to smartphone addiction among adolescents. Problem-focused coping strategies indicate that coping behaviors directly target the source of stress and can prompt participants to use positive coping styles to deal with the adverse consequences of the pandemic; conversely, coping styles of avoidance, denial, and fantasy in dealing with stress make it a potentially strong risk factor for smartphone addiction [65,66].

Limitations

This study had several limitations. The convenience sample limits the universality of the results. Factors such as family environment, personality traits, peer relationships,

and sleep quality may also affect mobile phone addiction among adolescents. Therefore, future studies should examine whether the relationship between Chinese adolescent resilience, coping style, DASS, and mobile phone addiction will change over time.

Conclusions

This study explored the impact mechanism of the effect of resilience on mobile phone addiction among Chinese adolescents .The main aim of the study was to establish if there was a relationship between resilience and mobile phone addiction in Chinese adolescents and if negative coping and DASS were a chain mediator of this relationship. The structural equation model was utilized to synchronously examine the individual and continuous mediating roles of coping styles and DASS. The results of indicate that a negative relationship exists between resilience and mobile phone addiction in this population. In addition, stress, anxiety, depression, and coping style significantly influence the risk of adolescent mobile phone addiction and play an intermediary role in Chinese adolescent resilience and mobile phone addiction. These results indicate the importance of mobile phone addiction and the importance of resilience for adolescents.Other studies have also shown that smartphone addiction caused health problems ， such as eating, sleeping,and mood disorder[67]. The findings may also help educators and medical personnel distinguish between predictive factors for adolescent mobile phone addiction. They could also be used to design an intervention to effectively treat and prevent mobile phone addiction in

adolescents when dealing with future difficult and traumatic events.At the same time, it also indicates the importance of cultivating the psychological resilience of adolescents in coping with pressure and difficulties since having good psychological resilience can reduce depression, anxiety, and reduce mobile phone addiction.

List of abbreviations

DASS: depression, anxiety, and stress; MPAI: mobile phone addiction index; RSCA: the Resilience Scale for Chinese Adolescents ; SCSQ: the Simplified coping style questionnaire.

Acknowledgements

Not applicable.

References

1.Lin YH, Chang LR, Lee YH, Tseng HW, Kuo TB, Chen SH. Development and validation of the Smartphone Addiction Inventory (SPAI). PLoS One. 2014 ;9(6):e98312. doi: 10.1371/journal.pone.0098312.

2. China Internet Network Information Center. https://www.cnnic.net.cn/hlwfzyj/hlwxzbg/hlwtjbg/202109/P020210915523670981527.pdf

3. Patton GC, Sawyer SM, Santelli JS, et al. Our future: a Lancet commission on adolescent health and wellbeing.Lancet.2016;387:2423-2478.

4. Ozamiz-Etxebarria, N., Dosil-Santamaria, M., Picaza-Gorrochategui, M., & Idoiaga-Mondragon, N. Stress, anxiety, and depression levels in the initial stage of the COVID-19 outbreak in a population sample in the northern Spain. Cadernos de Saude Publica.2020;36(4):e00054020. doi:org/10.1590/0102-311X00054020

5.Guang-Li Lu,Yue-Ming Ding,Yi-Ming Zhang, Hai-Tao Huang, Yi-Pei Liang, Chao-Ran Chen. The correlation between mobile phone addiction and coping

style among Chinese adolescents: a meta-analysis. Child and Adolescent Psychiatry and Mental Health.2021;15:60 . doi:org/10.1186/s13034-021-00413-2

6.The Tenth Press Conference of Henan Province Flood Control and Disaster Relief. Available online: http://www.henan.gov.cn/2021/08-02/2194036.html (accessed on 27 August 2021).

7. Enoka Randeniya. Flooding disaster: The effect on the adolescents at Angoda RahulaCollege, Sri Lanka. Procedia Engineering.2018;723–728.

8. Makwana N. Disaster and its impact on mental health: A narrative review. J Family Med Prim Care .2019;8:3090-5. doi:10.4103/jfmpc.jfmpc_893_19.

9.Agnew, R. (1992). Foundation for a general strain theory of crime and delinquency.Criminology.1992;30(1): 47–88. doi.org/10.1111/j.1745-9125.1992.tb01093.x

10.Jun, S., & Choi, E. (2015). Academic stress and Internet addiction from general strain theory framework. Computers in Human Behavior.2015;49:282–287. doi:10.1016/j.chb.2015.03.001

11.Greco R, Curci A. Does the General Strain Theory Explain Gambling and Substance Use? J Gambl Stud. 2017 ;33(3):919-936. doi: 10.1007/s10899-016-9654-2.

12.Mubarak, A. and Quinn, S. . General strain theory of Internet addiction and deviant behaviour in social networking sites (SNS), Journal of Information, Communication and Ethics in Society. 2019;17(1):61-71. doi.org/10.1108/JICES-08-2016-0024

13.Kim, Chaeyoon & Kwak, Keumjoo & Kim, Yeonsoo. The Relationship between Stress and Smartphone Addiction among Adolescents: The Mediating Effect of Grit. 2021. doi: org/10.21203/rs.3.rs-159399/v1

14.Tingting Gao, Jiaomeng Li, Han Zhang, Jinglei Gao, Yixi Kong, Yueyang Hu, Songli Mei.The influence of alexithymia on mobile phone addiction: The role of depression, anxiety and stress,Journal of Affective Disorders.2018;225:761-766. doi:org/10.1016/j.jad.2017.08.020.

15.Chih Hung Ko, Ju Yu Yen, Cheng Fang Yen, Cheng Sheng Chen, Chih Chi Weng, Cheng Chung Chen.The Association between Internet Addiction and Problematic Alcohol Use in Adolescents: The Problem Behavior Model.2008;11(5), 571-576. doi:org/10.1089/cpb.2007.0199

16.Makwana N. Disaster and its impact on mental health: A narrative review. J Family Med Prim Care. 2019 ;8(10):3090-3095. doi: 10.4103/jfmpc.jfmpc_893_19.

17.Ide-Okochi, A.; Samiso, T.;Kanamori, Y.; He, M.; Sakaguchi, M.;Fujimura, K. Depression, Insomnia,and Probable Post-Traumatic Stress Disorder among Survivors of the 2016 Kumamoto Earthquake and Related Factors during the Recovery Period Amidst the COVID-19 Pandemic. Int. J. Environ. Res. Public Health.2022;19:4403. doi:org/10.3390/ijerph19074403

18.Esterwood, E., Saeed, S.A. Past Epidemics, Natural Disasters, COVID19, and Mental Health: Learning from History as we Deal with the Present and Prepare for the Future. Psychiatr Q.2020;91:1121–1133. doi:org/10.1007/s11126-020-09808-4

19.Carli V, Durkee T, Wasserman D, Hadlaczky G, Despalins R, Kramarz E, et al. The association between pathological internet use and comorbid psychopathology: a systematic review. Psychopathology .2013;46:1-13.

20.Robert Fuchs, Maurya Glaude, Tonya Hansel, Joy Osofsky & Howard Osofsky. Adolescent risk substance use behavior, posttraumatic stress, depression, and resilience: Innovative considerations for disaster recovery, Substance Abuse.2021;42(3):358-365.doi: 10.1080/08897077.2020.1784357

21.Vella SL, Pai NB. A theoretical review of psychological resilience: Defining resilience and resilience research over the decades. Arch Med Health Sci 2019;7:233-9.doi:10.4103/amhs.amhs_119_19

22.Schriber, R. A. & Guyer, A. E. Adolescent neurobiological susceptibility to social context. Dev. Cogn. Neurosci.2016;19:1–18.

23.Harms, P. D., Brady, L., Wood, D., & Silard, A.. Resilience and well-being. In E. Diener, S.Oishi, & L. Tay (Eds.), Handbook of well-being. Salt Lake City, UT: DEF Publishers 2018.doi:nobascholar.com

24.Shen X. (2020) Is psychological resilience a protective factor between motivations and excessive smartphone use? Journal of Pacific Rim Psychology.2020; 14: e17. doi:org/10.1017/prp.2020.10

25.Davis SJ, Spillman S. Reasons for drug abstention: a study of drug use and resilience. J Psychoactive Drugs .2011;43:14-19.

26.Liu, Q.-Q., Zhou, Z.-K., Yang, X.-J., Kong, F.-C., Niu, G.-F., & Fan, C.-Y.. Mobile phone addiction and sleep quality among Chinese adolescents: A moderated mediation model. Computers in Human Behavior.2017; 72:108–114. doi:10.1016/j.chb.2017.02.042

27.Chen, L., Yan, Z., Tang, W., Yang, F., Xie, X., & He, J. . Mobile phone addiction levels and negative emotions among Chinese young adults: The mediating role of interpersonal problems. Computers in Human Behavior.2016;55:856-866.doi:10.1016/j.chb.2015.10.030

28.Dong, G. S., Park, Y., Min, K. K., & Park, J. . Mobile phone dependency and its impacts on adolescents' social and academic behaviors. Computers in Human Behavior.2016;63:282–292.doi:10.1016/j.chb.2016.05.026

29. Lazarus, R. S., and Folkman, S. Stress, Appraisal, and Coping. New York,NY: Springer1984.

30. Chapman, Paula L.; Mullis, Ronald L. Adolescent Coping Strategies and Self-Esteem. Child Study Journal.1999;29,1: 69-77.

31. Orgilés M, Morales A, Delvecchio E,et al. Coping Behaviors and Psychological Disturbances in Youth Affected by the COVID-19 Health Crisis. Front.Psychol. 2021;12:565-657.doi:org/10.3389/fpsyg.2021.565657

32.Fang Liu, Zhonghao Zhang, Liang Chen.Mediating effect of neuroticism and negative coping style in relation to childhood psychological maltreatment and smartphone addiction among college students in China.Child Abuse & Neglect.2020;106. doi:org/10.1016/j.chiabu.2020.104531.

33.de Terte, I., & Stephens, C. Psychological resilience of workers in high- risk occupations (Editorial). Stress and Health: Journal of the International Society for the Investigation of Stress.2014;30,5: 353–355. doi:org/10.1002/smi.2627

34.Li J, Chen Y-p, Zhang J, Lv M-m,Välimäki M, Li Y-f, Yang S-l, Tao Y-x,e B-y, Tan C-x and Zhang J-p.The Mediating Role of Resilience and Self-Esteem Between Life Events and Coping Styles Among Rural Left-Behind Adolescents in China: A Cross-Sectional Study.Front. Psychiatry .2020;11:560556.doi: 10.3389/fpsyt.2020.560556

35. Wu Y, Sang Z, Zhang X-C, Margraf J.The Relationship Between Resilience and Mental Health in Chinese College Students: A Longitudinal Cross-Lagged Analysis. Front. Psychol.2020;11:108.doi: 10.3389/fpsyg.2020.00108

36. Smida, M., Khoodoruth, M. A. S., Al-Nuaimi, S. K., et al. Coping strategies, optimism and resilience factors associated with mental health outcomes among medical residents exposed to coronavirus disease 2019 in Qatar. Brain and Behavior.2021;11:e2320. doi:org/10.1002/brb3.2320

37. Lovibond, P.F.; Lovibond, S.H. The structure of negative emotional states: Comparison of the Depression Anxiety Stress Scales (DASS) with the Beck Depression and Anxiety Inventories. *Behav. Res. Ther*.1995;*33*:335–343.

38. Louis Leung. Linking psychological attributes to addiction and improper use of the mobile phone among adolescents in Hong Kong. Journal of Children and Media.2008;2(2):93-113. doi:10.1080/17482790802078565

39. Yueqin Hu,Yiqun Gan. Development and validity of Resilience Scale for Chinese Adolescent. Acta Psychologica Sinica.2008;40(8)：902-912.

40.Ya-Ning Xie. A preliminary study on the reliability and validity of the Simplified Coping Style questionnaire. Chinese Journal Clinical Psychology.1998;6(2):114-115.

41.Baron,R.M.,Kenny,D.A.. The moderator–mediator variable distinction in social psychological research: Conceptual, strategic, and statistical considerations. Journal of Personality and Social Psychology.1986;51(6):1173-1182.

42. Hu, L. T., and Bentler, P. M.. Cutoff criteria for fit indexes in covariance structure analysis: conventional criteria versus new alternatives. Struct. Equ. Model.1999; 6(1):1–55.

43.Kline RB. Principles and Practice of Structural Equation Modeling. 2nd ed. New York, NY: Guildford Press;2005.

44..Kumpfe K.L. and Bluth B. Parent/child transactional processes predictive of resilience or vulnerability to “substance abuse disorders”. Substance Use & Misuse.2004;39: 671–698.

45. Mark Griffiths. A ‘components’ model of addiction within a biopsychosocial framework. Journal of Substance Use.2005;10(4): 191–197.

46. Lee Y.K., Chang C.T., Lin Y. and Cheng Z.H. The dark side of smartphone usage: Psychological traits, compulsive behavior and technostress.

Computers in Human Behavior, 2014； 31, 373–383.

47. Hou X.L., Wang H.Z., Guo C., Gaskin J., Rost D.H. and Wang J.L.

Psychological resilience can help combat the effect of stress on problematic social networking site usage. Personality & Individual Differences,2017； 109, 61–66.

48.Morales-Rodríguez FM, Martínez-Ramón JP, Méndez I and Ruiz-Esteban C .Stress, Coping, and Resilience Before and After COVID-19: A Predictive Model Based on Artificial Intelligence in the University Environment. Front. Psychol. 2021;12:647964. doi: 10.3389/fpsyg.2021.647964

49.Li J, Chen Y-p, Zhang J, Lv M-m, Välimäki M, Li Y-f, Yang S-l, Tao Y-x, Ye B-y, Tan C-x and Zhang J-p.The Mediating Role of Resilience and Self-Esteem Between Life Events and Coping Styles Among Rural Left-Behind Adolescents in China: A Cross-Sectional Study. Front. Psychiatry.2020;11:560556. doi: 10.3389/fpsyt.2020.560556

50.He A, Wang C, Hui Q. Loneliness and smartphone dependence: The role of negative coping. Chinese J Clin Psychol. 2018;26(6):1222 – 5.

51.Gao, T., Li, J., Zhang, H., Gao, J., Kong, Y., Hu, Y., & Mei, S. (2018). The influence of alexithymia on mobile phone addiction: The role of depression, anxiety and stress. Journal of Affective Disorders.2018; 225:761 – 766. doi:10.1016/j.jad.2017.08.020

52.Yang, X., Zhou, Z., Liu, Q. et al. Mobile Phone Addiction and Adolescents’ Anxiety and Depression: The Moderating Role of Mindfulness. J Child Fam Stud.2019;28:822–830. doi:org/10.1007/s10826-018-01323-2

53.Choksi S, Patel N. A Study to Find Out the Correlation of Mobile Phone Addiction with Anxiety, Depression, Stress and Sleep Quality in the College Students of Surat City. Int J Curr. 2021;13(08):137-142.

doi: http://dx.doi.org/10.31782/IJCRR.2021.13812

54.Lei, L. Y.-C., Ismail, M. A.-A., Mohammad, J. A.-M., & Yusoff, M. S. B. . The relationship of smartphone addiction with psychological distress and neuroticism among university medical students. BMC Psychology.2020;8(1):97. doi:10.1186/s40359-020-00466-6

55.Buabbas AJ, Hasan H, Buabbas MA.The associations between smart device use and psychological distress among secondary and high school students in Kuwait. PLoS ONE.2021;16(6): e0251479. doi:org/10.1371/journal. pone.0251479.

56.Stanković M, Nešić M. Association of internet addiction with depression, anxiety, stress, and the quality of sleep: Mediation analysis approach in Serbian medical students. Current Research in Behavioral Sciences.2022;3: 100071.

doi:org/10.1016/j.crbeha.2022.100071

57. Lu GL, Ding YM, Zhang YM, Huang HT, Liang YP, Chen CR. The correlation between mobile phone addiction and coping style among Chinese adolescents: a meta-analysis. Child Adolesc Psychiatry Ment Health.2021;15(1):60. doi: 10.1186/s13034-021-00413-2.

58.Ozen S, Topcu M. The relationship of smartphone addiction with depression, obsession-compulsion, impulsivity, alexithymia among medical faculty students. Journal of Dependence .2017;18:16-24.

59.Elhai JD, Tiamiyu MF, Weeks JW, Levine JC, Picard KJ, Hall BJ. Depression and emotion regulation predict objective smartphone use measured over one week. Pers Individ Dif .2018;133:21-28.doi:org/10.1016/j.paid.2017.04.051.

60. Pera A . The Psychology of Addictive Smartphone Behavior in Young Adults: Problematic Use, Social Anxiety, and Depressive Stress. Front. Psychiatry. 2020;11:573473. doi: 10.3389/fpsyt.2020.573473

61. Sonali Tanmay Choksi; Nipa Patel. A Study to Find Out the Correlation of Mobile Phone Addiction with Anxiety, Depression, Stress and Sleep Quality in the College Students of Surat City. Int J Cur Res Rev .2021;13(8):137-142.

doi: org/10.31782/IJCRR.2021.13812.

62. Al. Qudah, M.F.; Albursan,I.S.; Hammad, H.I.; Alzoubi, A.M.;Bakhiet, S.F.; Almanie, A.M.; Alenizi,S.S.; Aljomaa, S.S.; Al-Khadher, M.M.Anxiety about COVID-19 Infection,and Its Relation to Smartphone Addiction and Demographic

Variables in Middle Eastern Countries. Int. J. Environ. Res. Public Health.2021; 18: 11016. doi:org/10.3390/ijerph182111016.

63. Ahn, S. Y., Kim, Y. J. The influence of smart phone use and stress on quality of sleep among nursing students. Ind. J. Sci. Technol.2015;8:1–6. doi:10.17485/ijst%2F2015%2Fv8i35%2F85943

64. Einar B. Internet Addiction, Psychological Distress, and Coping Responses Among Adolescents and Adults. CYBERPSYCHOLOGY, BEHAVIOR, AND SOCIAL NETWORKING.2017;20(5):296-304. doi: 10.1089/cyber.2016.0669

65. Stahl GK, Caligiuri P. The effectiveness of expatriate coping strategies: the moderating role of cultural distance, position level, and time on the international assignment. J Appl Psychol.2005;90:603–15. doi: 10.1037/0021-9010.90.4.603

66. Duan L, He J, Li M, Dai J, Zhou Y, Lai F and Zhu G . Based on a Decision Tree Model for Exploring the Risk Factors of Smartphone Addiction Among Children and Adolescents in China During the COVID-19 Pandemic. Front. Psychiatry. 2021;12:652356. doi: 10.3389/fpsyt.2021.652356

67.Napassphol Sinsomsack,Waiphot Kulachai.A study on the impacts of smartphone addiction. Proceedings of the 15th International Symposium on Management.2018;186:248-252.doi:10.2991/insyma-18.2018.61

Supporting information

S1 Table.

Table 1: Demographic profiles and descriptive statistics of the participants.

**Gender**	Frequency	Percentage
boy	979	43.2
girl	1289	56.8
**One-child**		
yes	169	7.5
no	2099	92.5
**Birth order**		
1st	1048	46.2
2nd	1041	45.9
3rd	179	7.9
**Nationality**		
Han	2259	99.6
Hui	8	0.4
Miao	1	0.0
**Grade**		
middle school 7th	174	7.7
middle school 8th	634	28.0
middle school 9th	436	19.2
high school 1st	34	1.5
high school 2nd	471	20.8
high school 3rd	519	22.9
Total	2268	100.0

S2 Table.

	M	Std.Error of Mean	Frequency	Percentage
**Age**	14.90	2.58		
**MPAI Total**	39.57	13.82		
feeling anxious&lost	7.96	4.02		
inability to control craving	16.65	5.92		
productivity loss	7.64	3.28		
withdrawal	7.32	3.49		
**DASS-21 Total**	5.19	4.57		
**Stress**	7.40	5.07		
normal			2054.00	90.56
mild			124.00	5.47
moderate			76.00	3.35
severe			14.00	0.62
extremely severe			0.00	0.00
**Anxiety**	6.31	4.94		
normal			1583.00	69.80
mild			218.00	9.61
moderate			304.00	13.40
severe			103.00	4.54
extremely severe			60.00	2.65
**Depression**	6.58	5.09		
normal			1773.00	78.18
mild			258.00	11.38
moderate			179.00	7.89
severe			48.00	2.12
extremely severe			10.00	0.44
**SCSQ Total**	56.39	17.91		

positive coping	22.45	9.18
negative coping	11.93	5.64
**RSCA Total**	88.89	18.50
focuced	16.24	4.88
Interpersonal support	18.51	5.90
emotional control	19.63	5.76
Positive cognitive	14.30	3.78
familiy support	16.77	4.50

Table 2. Basic characteristics and measure scores.

S3 Table.

Table3: Correlation analysis of study variables

	**1**	**2**	**3**	**4**
**1.RSCA Total**	1			
**2.Negative coping**	-.317**	1		
**3.DASS-21 Total**	-.593**	.603**	1	
**4.MPAI Total**	-.405**	.322**	.448**	1

Note: $^{**}P < 0.01$

S4 Table.

Table 4. Results of the structural model: tests of hypothesized associations between constructs.

			**Estimate**	**S.E.**	**t-value**	**P**
**negative coping**	**<---**	**RSCA**	-1.025	0.078	-13.216	***
**DASS**	**<---**	**negative coping**	0.292	0.017	16.871	***
**DASS**	**<---**	**RSCA**	-1.152	0.061	-18.992	***

**MPAI**	<---	**RSCA**	-0.836	0.07	-11.919	***
**MPAI**	<---	**negative coping**	0.09	0.017	5.355	***
**MPAI**	<---	**DASS**	-0.096	0.031	-3.092	0.002

Note: ***P < 0.01

S5 Table.

Table 5. Bootstrap truncated regression results.

				**Bootstrapping**				
	**Product of coefficients**			**BC 95%CI**		**Percentile 95%CI**		
Relationships	**point estimate**	***SE***	***Z***	**Lower**	**Upper**	**Lower**	**Upper**	***P***
				Indirect Effects				
**RSCA → negative coping → MPAI**	-0.092	0.018	-5.111	-0.125	-0.061	-0.125	-0.061	0
**RSCA → DASS → MPAI**	0.111	0.043	2.581	0.043	0.188	0.045	0.186	0.002
**RSCA → negative coping → DASS→ MPAI**	0.029	0.011	2.636	0.012	0.048	0.012	0.048	0.002
**Total**	-0.789	0.052	-15.173	-0.889	-0.698	-0.89	-0.699	0

Figure2

Click here to access/download;Figure;Figure_2.The standardized path coefficients.tiff

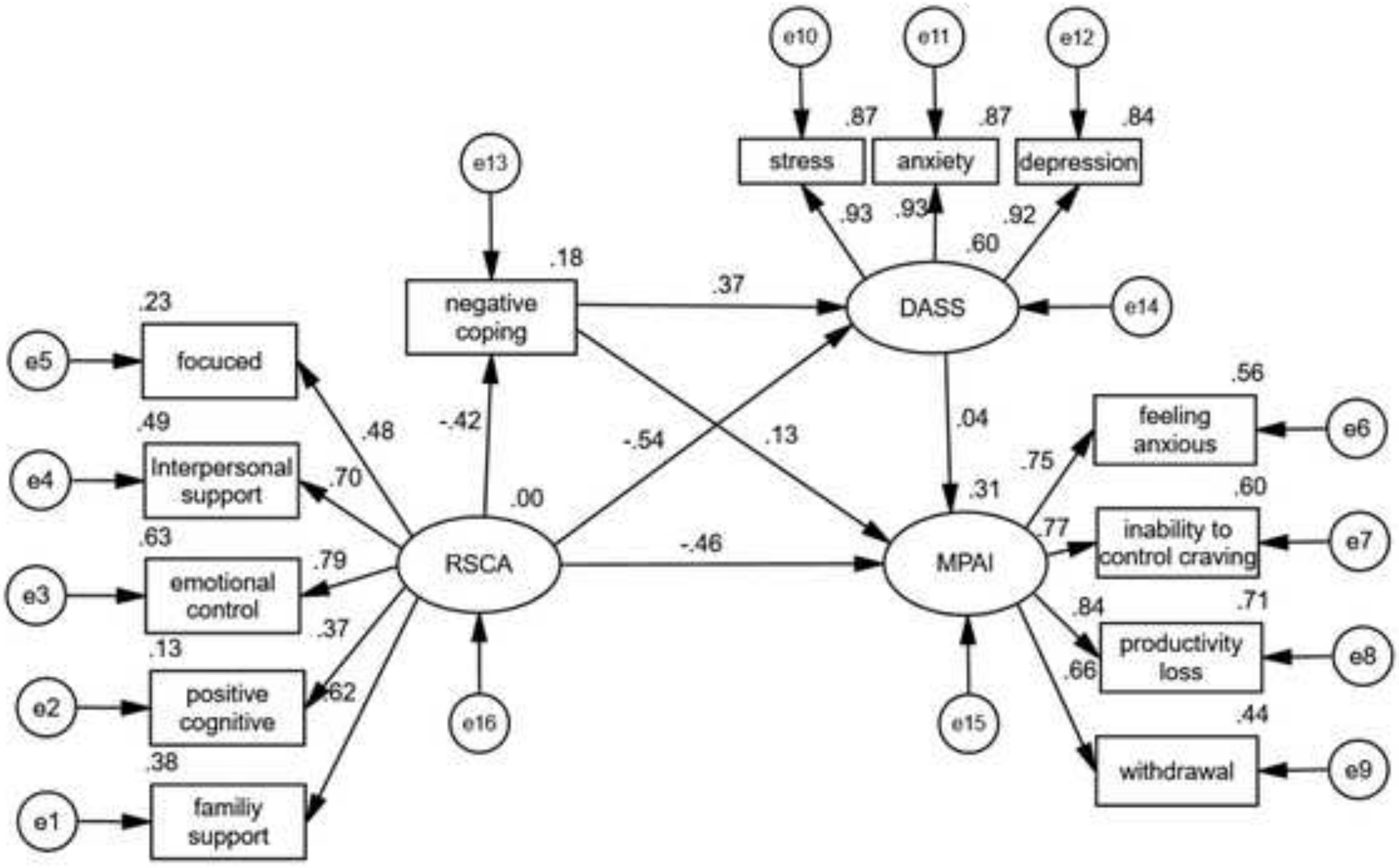

Figure 2 The standardized path coefficients in model testing

Figure1

Click here to access/download;Figure;Figure 1 Hypothesized model.tiff

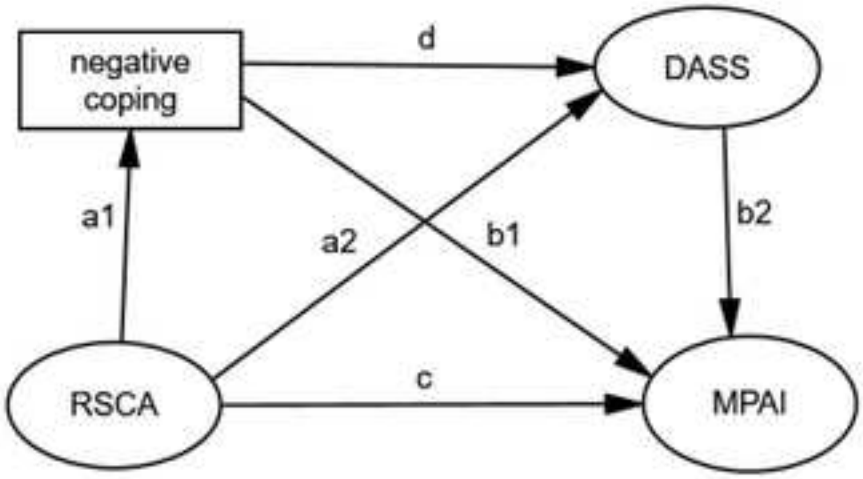

Figure 1 Hypothesized model

Notes: mobile phone addiction index (MPAI); depression, anxiety, and stress scale with 21 items (DASS-21); Resilience Scale for Chinese Adolescents (RSCA); Simplified coping style questionnaire (SCSQ).

Click here to access/download
Supporting Information
tables.docx

Click here to access/download
Supporting Information - Compressed/ZIP File Archive
data.xlsx

Adolescent Resilience and Mobile Phone Addiction in Henan ~~p~~Province of China: Impacts of Chain Mediating, Coping Style

Anna Ma[1 #a #b], Yan Yang[1], Shuangxi Guo[2], Xue Li[1], Shenhua Zhang[3], Hongjuan Chang[1*]

[1 #a] Xinxiang Medical University~~-~~, School of Nursing, Xinxiang, Henan province,China

[#b] St. Paul University, School of Nursing, Manila, Philippines

[2]The First Affiliated Hospital of Xinxiang Medical University, Department of Neurology, Xinxiang, Henan province, China

[3] Weihui Senior Middle School, Xinxiang, China

*** Correspondence:**

Hong ~~j~~Juan Chang

E-mail: changhj0812@126.com

Conflict of Interest/Disclosure Statement

Competing interests

The authors declare that they have no competing interests and all methods were carried out in accordance with relevant guidelines and regulations. The authors alone are responsible for the content and writing of the article.

Author Contributions

Hong Juan Chang designed the study, Xue Li and Shenhua Zhang collected the data, Anna Ma analyzed the data and wrote the manuscript. Yan Yang and Shuangxi Guo revised the manuscript. All authors contributed to the article and approved the submitted version.

Formatted: Level 1, Space Before: 12 pt

~~**Funding**~~

~~This work was supported by the National Natural Science Foundation of China(grant number: 81803252).~~

~~**Ethics approval and consent to participate**~~

~~The participants were under 18 years old, and we provided informed consent to their parents (or legal guardians). All procedures performed in studies involving human participants were approved by the Ethics Committee of Xinxiang Medical University(#XYLL-2018015).~~

Acknowledgements

Not applicable.

Abstract

Formatted: Font: 18 pt

Background: As mobile phone use grows, so it brings benefits and risks. As an important part of adolescents healthy growth, resilience plays an indispensable role ~~in Chinese adolescents~~. Thus, it is important to identify when mobile phone use of an adolescent ~~use is~~becomes an addiction. This study proposed to explore the effects of adolescent resilience on mobile phone addiction, and tested the mediating role of coping style and depression, anxiety, and stress (DASS) on phone addiction among 2,268 adolescents in the Henan ~~P~~province,China.

Methods: The ~~–~~adolescents were surveyed via an online questionnaire,a mobile phone addiction index (MPAI), a depression, anxiety, and stress scale with 21 items (DASS-21), the Resilience Scale for Chinese Adolescents (RSCA), and the Simplified coping style questionnaire (SCSQ), and we used structural equation modeling to examine the correlations and moderation effects. All data analyses were performed using SPSS 26.0 and Amos 23.0.

Results: The results show that adolescences resilience were negatively related to negative coping ,DASS, and mobile phone addiction;~~–~~both coping style and DASS could mediate the relationship between adolescent resilience and mobile phone addiction among Chinese adolescents. The relationship between adolescent resilience and mobile phone addiction in Chinese adolescents was mediated by the chain of coping styles and DASS.

Conclusions: There is a negative relationship which exists between resilience and mobile phone addiction in this population. In addition, stress, anxiety, depression, and

coping style significantly influence the risk of adolescent mobile phone addiction and play an intermediary role in Chinese adolescent resilience and mobile phone addiction.

Keywords: Adolescent resilience; Ccoping style; Mmobile phone addiction; China; DASS-21; Cchain mediating

Introduction

Formatted: Font: 18 pt, (Asian) Chinese (PRC)

Formatted: Font: Times New Roman, 18 pt

Formatted: Font: 18 pt, (Asian) Chinese (PRC)

With the development of information technology, the functions of a mobile phone isare becoming more and more powerful,; it is now considered as an important communication tool and social accessory.However,increased dependency brings a downside, namely,smartphone phone addiction.Smartphone addiction consists of four main components: obsessive phone use and compulsive behaviors; tolerance of longer and more intense of use; withdrawal or suffering without the phone; and functional impairment [1].

–Due to the COVID-19 outbreak, mobile phones have become increasingly important for online teaching and learning in China. According to the 48th China statistical report on internet development, by June 2021, there were 1.07 billion mobile phone users in China, accounting for 99.7% of the total number of internet users. Furthermore, internet users spend an average of 26.9 hours online per week, and the number of internet users between the ages of 6 and 19 reached 158 million, accounting for 15.7% of the total (China Internet Network Information Center,2021)[2]. As part of normal adolescent psychological development, this age group develops susceptibility to peer influences and tends to have low-risk perception—factors that can result in increased risk-taking

behavior and poor self-regulation_ (Patton GC,2016)[3]. Stressful life events have been identified as a significant risk factor in the emotional state of adolescents. TheRegarding the emotional state of adolescents, stressful life events have been identified as a significant risk factor, and the spread of COVID-19 has been one of these stressful life events as it led to substantial social and economic changes as a result of because many governments introducing adapted quarantine policies to prevent the virus from spreading and thus assure the safety of the population.to assure a population of safety and prevent the virus from spreading. Psychopathologists have focusedfocused on the psychological impact of COVID-19 and its variations in the adolescent population and they have identified; they identified moderate to severe levels of stress, anxiety, and depression in this population (Ozamiz-Etxebarria, N.,2020)[4]. There was Guang-Li found that a moderate positive correlation between negative coping style and adolescents' mobile phone addiction (Guang Li Lu,2021)[5].

In the middle of July 2021, the Henan province suffered from unusually heavy rainfall and maximum (continuous rainfall of 958 mm (The Tenth Press Conference of Henan Province Flood Control and Disaster Relief,2021)[6]), causing severe flooding. The flood, named the "7.20 Henan rainstorm," blasted the overwhelmed dams and burst riverbanksbanks of rivers in a short time, causing severe traffic paralysis, water power failure, loss of crops,waterlogging, and upending tens of millions of lives. Randeniya reported that after this natural disaster, adolescents of Sri Lanka were mostly affected by sleeping difficulties sleeping difficulties were the most affected problem by flooding disasters in adolescents of Sri Lanka (Enoka Randeniya,2018)[7]. Makwana indicated

that the psychological effects of the disaster were fiercer among children, women, and the dependent elderly population ~~(Makwana N,2019)~~[8].

The general strain theory argues that adolescents can be pressured into delinquency by a negative experience,this negative effect creates pressure for corrective action and may lead adolescents to escape from the source of their adversity, or manage the negative effect through the use of illicit drugs[9]. The general strain theory was originally used for adolescents' criminal behavior, but now many researchers use it for explaining addictive behaviors [10-12].Recent studies have argued that stress and disaster can cause mobile phone addiction through various pathways[13-15].Disaster can lead to severe stress,uncontrollable stress,and substance dependency of the individual[16].According to many studies, natural disasters and COVID-19 as stressors can lead people to depression, anxiety, substance abuse,and addictive behavior[17,18].Some experts also believe that depression, anxiety, and pressure may lead to internet addiction[19].Research has also shown that exposure to disaster is positively associated with substance abuse and is negatively related to children's psychological resilience[20]. Resilience is commonly described as the ability to bounce back or overcome some form of adverse event or situation and thus experience positive outcomes [21].Resilience is an important developmental stage during adolescence, as it is a transitional period characterized by significant neurobiological and psychosocial changes in the context of amplifying environmental demands and increasing sensitivity to social contexts [22].One study has found that there is a negative relation between high psychological resilience and depression[23].Resilience is also a protective factor of excessive

smartphone use[24].Many scholars believe that adolescent resilience is also related to substance abuse, such as smoking and excessive drinking (alcoholism) [25]. However, studies on the relationship between mental resilience and adolescents' coping styles, mental health, and mobile phone addiction are rare.

Furthermore, research has shown significant differences exist in psychological effects among adolescents in terms of gender and family size(Satadeepa Som,2019; Breik,2019).

Many scholars believe that adolescent resilience is also related to substance use, such as smoking and excessive drinking (alcoholism) (Davis SJ,2011). Some experts also believe that depression, anxiety, and pressure may lead to internet addiction(Carli V,2013). In view of this, adolescents confined to their homes due to the floods in Henan may have overused their mobile phones and the internetHowever, studies on the relationship between mental resilience and adolescents' coping styles, mental health, and mobile phone addiction are rare.

In view of this, adolescents confined to their homes may be likely to overuse mobile phones and the internet due to floods in Henan province of China. Many studies have shown that mobile phone addiction has had a negative impact on physical and mental health as well as social adaption,academic achievement, and sleep quality[26-28].Thus, we hypothesized that there is a correlation between resilience and mobile phone addiction among adolescents and that coping style and mental health play a mediating role in that relationship.

"Coping" means constantly changing cognitive and behavioral efforts to manage

specific external and/or internal demands that are appraised as taxing or exceeding a person's resources ~~(Lazarus, R. S.,1984)~~[29]. Chapman thought that adolescents with lower self-esteem engage in coping strategies of ventilating feelings, avoiding problems, and relaxing, while~~and~~ adolescents with higher self-esteem were more likely to engage in coping styles that directly address solving the problem~~(Chapman, Paula L.1999)~~[30]. During the COVID-19 pandemic, children who used positive strategies to cope with the situation suffered less emotionally and behaviorally ~~(Orgilés M,2021)~~[31]. Fang Liu found that a negative coping style mediated the relationship between smartphone addiction and childhood psychological maltreatment~~(Fang Liu,2020)~~[32]. Psychological resilience is the ability to cope with a crisis or to quickly return to a pre-crisis status, either mentally or emotionally [33]~~Psychological resilience is the ability to cope with a crisis or to quickly return to a pre-crisis status from mentally or emotionally (de Terte, I., & Stephens, C,2014)~~.

~~Resilience is an important developmental stage during adolescence, as it is a transitional period characterized by significant neurobiological and psychosocial changes in the context of amplifying environmental demands and increasing sensitivity to social contexts (Schriber, R. A. & Guyer, A. E,2016).~~

~~Resilience has neurobiological, physical, social, cognitive-behavioral, and emotional regulation foundations (Southwick, S. M.,2014; Southwick, S. M. & Charney, D. S,2012; Hu, T., Zhang, D. & Wang, J,2015). However, how the neurobiological and psychosocial factors influence resilience in adolescence is not well understood. Malhi et al. proposed a model that captures the dynamic nature of resilience in adolescents,~~

Formatted: Font: (Default) Times New Roman, 12 pt

Formatted: Font: (Default) Times New Roman, 12 pt

Formatted: Font: (Default) Times New Roman, 12 pt, Not Italic

with special attention to how it emerges, how neurobiological and psychosocial factors interact to build and strengthen resilience, and individual differences in resilience (Malhi, G.S., 2019). Resilience negatively predicted thea negative coping style and positively predicted thea positive coping style,. lLife events not only directly had direct influenced a negative coping style and positive coping style but also indirectlyhad indirect influenced coping styles by affecting resilience(Li J,2020)[34]. The mutually enhancing relationship between resilience and positive mental health, and vice versa, a mutually reducing relationship between resilience and mental illness, and presented the significant influence of mental health level on resilience(Wu Y,2020)[35]. Malek showed that the coping style of avoidance couldavoidant coping styles can aggravate depressive, anxiety, and stress symptoms of depression, anxiety, and stress in participants during the COVID- 19 pandemic. Keeping optimism, resilience, and approach coping styles decreasedcan decrease the mental health burden of the pandemic on participants (Smida, M.,2021)[36].

The present study

Formatted: Font: 18 pt, Bold

Based on the literature review above, the relationship between adolescent resilience and mobile phone addiction deserves attention. tThe present study constructed a chain mediation model to examine the mediating role of negative coping, stress, anxiety, and depression in the relationship between adolescent resilience and mobile phone addiction among Chinese adolescents. Furthermore, we proposed a model to test the associations among Chinese adolescent resilience, coping style, mental health, and mobile phone addiction, to further clarify mobile phone addiction related to resilience

(Fig 1~~ure 1~~).

Materials and Methods

Formatted: Font: 18 pt

Formatted: Font: 18 pt, Bold

Participants

The convenience sampling method was employed to select adolescents~~students~~ from ~~grades 7th to 9th of~~ middle school and ~~grades 1st to 3rd of~~ high school in Henan province of China to complete the questionnaires online from July 1 to August30, 2021. In total, 2,268 valid questionnaires were obtained, with an effective rate of 97.28%. Among them, the mean age was 14.90 years (*SD* = 2.58, range = 12–21 years), participants including~~included~~ 979 boys(43.20%)and 1,289 girls (56.80%). They completed a survey that included demographic variables, a mobile phone addiction index (MPAI), a depression, anxiety, and stress scale with 21 items (DASS-21), the Resilience Scale for Chinese Adolescents (RSCA), and the Simplified ~~c~~Coping ~~s~~Style questionnaire (SCSQ).The link to the questionnaires was sent out to potential participants. All questions were completed by respondents clicking on the appropriate option.In the case of a missing answer, the server would remind them to give the missing answer. All participants filled in the questionnaire anonymously, without any payment.Their privacy was protected.

Ethics approval and consent to participate

The participants were under 18 years old, and we provided written informed consent to their parents (or legal guardians)before taking part in the study . The procedures performed in the study were approved by the Ethics Committee of Xinxiang Medical

University(number:XYLL-2018015),as are all studies involving human participants. The data from the participants were de-identified.

Measurement of Structures

DASS-21

The DASS-21was used to evaluate negative emotional states of depression, anxiety, and stress~~(Lovibond, P.F., Lovibond, S.H,1995)~~[37] and~~,~~ refer to the previous week.~~,~~ In the DASS-21,~~and~~ each item is classified into four Likert responses from 0 to 3, where ~~from~~ 0 = “nothing” and~~to~~ 3 = “Most of the time.” This self-report instrument includes three subscales: 1) the stress subscale, which measures tension, agitation, difficulty relaxing, and negative affection; 2)the anxiety subscale, which assesses autonomic arousal, skeletal musculature effects, situational anxiety, and subjective experience of anxiety arousal~~anxious arousal~~; and 3) the depression subscale, which measures hopelessness, dysphoria, lack of interest, self-deprecation, and inertia. The reliability coefficients of depression, anxiety and stress were 0.82, 0.82 and 0.79, respectively. The Cronbach’s alpha of the total scale was 0.89.

Formatted: Font: (Default) Times New Roman, 12 pt, Not Italic

MPAI

The MPAI was designed by Louis Leung to identify addiction symptoms associated with mobile phone use among adolescents in Hong Kong~~(Louis Leung,2008)~~[38]. The scale includes 17 items answered on a five-point Likert scale of 1 to 5 (1 = not at all; 2 = rarely; 3 = occasionally; 4 = often; and 5 = always). The scale covers four

dimensions: 1) “inability to control craving,” which reflects the amount of time an adolescent spends adolescents spend on the mobile phone, thereby leading to complaints from family and friends about their compulsive mobile phone use and causing the adolescent’sadolescents loss of sleep due to the excessive use; 2) “Anxiety and feeling lost” assesses preoccupation, feeling lost or anxious, and having difficulty switching off the mobile phone; 3)“Prproductivity loss” measures decreased productivity and diverted attention from pressing issues due to an adolescent’sadolescents’ excessive use mobile phones; 4) “wWithdrawal and escape” indicates that an adolescents uses their mobile phones to escape from isolation, loneliness, and feeling down. The Cronbach’s alpha of scale was 0.90.

RSCA

The RSCA was developed by Yueqin Hu(Yueqin Hu, 2008)[39]according to the process model of the resilience concept and applied to Chinese adolescents. There are 27 items divided into two factors: “manpower” and “support”;. tThe former includes three factors: goal focus, emotion control, and positive cognition ; thewhile latter includes two factors: family support and interpersonal assistance. The reliability of the total scale was 0.85.

SCSQ

This SCSQ was designed by Ya-Ning Xie (Ya-Ning Xie,1998)[40], combined with the characteristics of culture in China, to simplify the ways of coping questionaires and modify, compiled a simple coping style questionnaire, composed of 20

items, Coping styles can be divided into two groups based on how people react to setbacks often involving people in daily life may take different attitude and measures, according to people in relatively separated suffered setbacks coping styles can be two categories: "positive coping" and "negative coping." The reliability of the total scale was 0.90, while the positive coping and negative response subscales were 0.89 and 0.78, respectively.

Formatted: Font: (Default) Times New Roman, 12 pt, Font color: Auto

Data Analysis

All data analyses were performed using SPSS 26.0 and Amos 23 (IBM Inc., Armonk, NY, USA). First, descriptive data were received using SPSS 26.0, and correlations variables were calculated using Pearson's correlations. Second, according to Baron and Kenny(Baron,R.M.,Kenny,D.A. ,1986)[41], we analyzed the mediation effects using two measurement models to examine how well the indicators represented each latent variable. Second, we tested the hypothesized relationships among latent variables. Maximum likelihood (ML) estimation was used to test the two structural models in the AMOS 23.0 program. When TLI > 0.90, CFI > 0.90, and RMSEA < 0.06, the model fits well, according to Hu and Bentler (Hu, L. T., 1999)[42].We followed the stepwise method to structure the best-fitting model for the mediated effects and bootstrapping with 5,000 replications to measure the chain mediation model. All data analyses were two-tailed, with significance levels of $P < 0.01$ and $P < 0.05$.

Results

Formatted: Font: 18 pt

Descriptive Statistics

We included 2,268 participants, including 979 boys (43.20%) and 1,289 girls (56.80%), in the final analysis. The proportion of girls was slightly higher than that of boys (56.80% vs. 43.20%). The mean age was 14.90 years (SD = 2.58, range 12–21 years). There were 1244 (54.85%) participants ~~in the~~from middle school, 1,024 (45.15%) ~~in the~~from high school, 169 (7.50%) from one-child families, and 2,099 (92.50%) from multi-child~~were from non-one-child~~ families. The other results are shown in Table 1.

Univariate Analysis

The results for the 2,268 participants are displayed in Table 2.~~As displayed in Table 2, the results for the 2268 participants,~~ The category totals as total mean (*SD*) ~~are~~were as follows: MPAI, 39.57 (±13.82); DASS-21, 5.190 (±4.57); positive coping was 22.45 (±9.18); negative coping, 11.93 (±5.64); RSCA, 88.89 (±18.50).

Correlation Analysis of Major Study Variables

The variables correlated with the constructs in Table 3 were less than 0.85. The discriminant validity value (< 0.85) was met in the construct correlation ~~(Kline RB,2005)~~[43]. These findings showed that valid and reliable constructs were used.We found that adolescents resilience were negatively related to negative coping ,DASS, and mobile phone addiction. Negative coping was positively related to both DASS

and mobile phone addiction. The results of analyses also showed that adolescent resilience correlated with negative coping (r = -.317, *P*< 0.01) , DASS(r =-.593,*P*< 0.01),and mobile phone addiction(r=-.405,*P*<0.01);negative coping correlated with DASS(r=.603,*P*<0.01),mobile phone addiction(r=.322,*P*<0.01);DASS correlated with mobile phone addiction(r=.448,*P*<0.01).Adolescents with poor resilience usually adopted a negative coping style and had higher anxiety, depression and mobile phone addiction—which confirms the hypothesis .

Formatted: Font: Italic

Formatted: Font: Italic

Formatted: Font: Italic

Formatted: Font: Italic

Formatted: Font: Italic

Formatted: Font: Italic

Structural Model Testing and Structural Relationship Between Constructs

The test results revealed the goodness of fit of the proposed structural model ($\chi 2$/df = 2.57, RMSEA = 0.054, GFI = 0.978, CFI = 0.984). The hypothesis relationships between the variates are demonstrated in Table 4 ~~and Figure 2~~. Results of the test for path(negative coping <---RSCA) revealed a significant negative relationship between resilience and negative coping (β = -1.025, t = -13.216, *P* < 0.01). Results of the test for path(DASS <---negative coping) showed a significant positive relationship between DASS and negative coping (β = 0.292, t = 16.871,*P* < 0.01). Results of the test for path(DASS <---RSCA) revealed a significant negative relationship between DASS and resilience (β = -1.152, t = -18.992, *P* < 0.01). Results of the test for path(MPAI <---RSCA)showed a significant negative association between mobile phone addiction and resilience (β = -0.836, t =-11.919, *P* < 0.01). Results of path(MPAI <---negative coping)showed a significant relationship positive relationship between mobile phone addiction and negative coping (β = 0.09, t =

Formatted: Font: Italic

Formatted: Font: Italic

Formatted: Font: Italic

Formatted: Font: Italic

5.355, *P* < 0.01). Results of the test for path(MPAI <---DASS)revealed a significant negative relationship between mobile phone addiction and DASS (β = -0.096, t = -3.092, *P* = 0.002). Finally, the results confirmed the hypothesis.The variables were explained by their respective preceding construct (Fig 2)The indirect effects are presented in Table 5. Bootstrapping (the process was repeated 5,000 times)analyses showed that the indirect effects of adolescent resilience on mobile phone addiction through negative coping and stress, anxiety, and depression were significant and positive (standardized indirect effect 0.029,95%CI [0.012,0.048], *P* < 0.01), and the indirect effect of adolescent resilience on mobile phone addiction through stress, anxiety, depression was 0.111, 95% CI [0.045, 0.186], *P* < 0.01, excluding 0, and the mediating effect was significant. The indirect effect of adolescent resilience on mobile phone addiction through negative coping was -0.092, 95% CI [-0.125, -0.061], *P* < 0.01, excluding 0, and the mediating effect was significant.

Discussion

This study surveyed the ways by which adolescent psychological resilience, coping style and ~~D~~depression, anxiety, and stress affected mobile phone addiction among Chinese adolescents.The main aim of the study was to establish if there was a relationship between resilience and mobile phone addiction in Chinese adolescents and if negative coping and DASS were a chain mediator of this relationship. –The results showed that adolescent psychological resilience could directly and negatively affect mobile phone addiction in Chinese adolescents, in consequence,poor resilience

Formatted: Font: Italic

Formatted: Font: Italic

Formatted: Font: 18 pt

may cause mobile phone addiction in Chinese adolescents,which is consistent with previous research findings (Robertson, T.W., 2018).

According to the psychological resilience framework theory (Kumpfe K.L. , 2004)[44], psychological resilience is an important protective factor for problem behavior and personal mental health. Griffiths argued that addictions consist of several components, such as relapse, mood modification, tolerance, conflict, and withdrawal (Mark Griffiths,2005)[45]. In the studies, psychological resilience was found to protect the addictive behaviors (internet problematic use)(Lee Y.K.,2014; Hou X.L.,2017)[46,47].Adolescent mobile phone addiction affect their life and study, and this study suggests that family, peer, teacher support, and exercise help to improveenhance the depressive moodsbrain and make it more resilient to adversity and stress.

The experimental results revealshow that adolescents psychological resilience negative coping style is negatively correlated with negative coping style adolescents psychological resilienc: e, negative coping style is negatively correlated with mobile phone addiction. It shows that adolescents with good psychological resilience are more likely to be adopt positive coping styles when facing pressure and frustration and are less likely to addicted to mobile phones.Francisco found that higher scores on perceived stress represented lower resilience and lower use of the coping strategies of problem-focusing[48].Li et al. [49]also found that resilience could negatively predict the negative coping style and positively predict the positive coping style ,which is

consistent with the results of this study.He et al. [50] found that adolescents adopt a negative coping style, which leads to mobile phone addiction. This study reveals that depression, anxiety, and stress (DASS)is negatively correlated with mobile phone addiction. Gao et al found that perceived stress was directly associated with mobile phone addiction[51].A large number of studies have shown that mobile phone addiction is positively associated with anxiety and depression,which in turn brings about severe adverse effects on mental health,such as anxiety ,depression and poor sleep quality[52-55].These studies suggest an interplay between anxiety, depression, and phone addiction.;

tThisese results show that negative coping styles and depression, anxiety, and stress (DASS) play intermediary roles, respectively, in adolescent resilience and mobile phone addiction in Chinese adolescents; thus, our hypothesis was verified. This finding is consistent with the results of previous studies.For example, Stanković found partial mediation between internet addiction and depression through stress and anxiety[56].

Understanding how one copes with stress and managing coping styles can be particularly effective for smartphone addiction (Alan R, 2020). The Simplified Coping Style Questionnaire(SCSQ) results revealed that coping style had a maximal effect on adolescent mobile phone addiction (Lu GL,2021)[57]. Many studies have indicated a relationship between depression, anxiety, and loneliness with smartphone usage (Ozen S, 2017; Elhai JD,2018)[58,59]. Depression and social anxiety are risk

determinants for greater problematic smartphone use ~~(Pera A,2020)~~[60]. Stress, anxiety, and depression were significantly positively correlated with smartphone addiction ~~(Sonali Tanmay Choksi, 2021)~~[61]. Researcher have found~~The found~~ that a significant positive relationship between anxiety about COVID-19 infection and daily smartphone use hours; the largest predictor of smartphone addiction was anxiety about COVID-19 infection ~~(Al. Qudah, M.F.,2021)~~[62].

Negative coping style and DASS played a continuous intermediary role in the impact of adolescent resilience on mobile phone addiction among Chinese adolescents. Smartphone users who experience depressive symptoms may similarly use their mobile devices as a coping strategy to alleviate these unpleasant symptoms ~~(Ahn, S. Y., 2015)~~[63]. Coping and affective disorders appear to play a key role~~key roles~~ in international addiction among adolescents ~~(Einar B,2017)~~[64]. COVID-19 and floods as stressors can cause psychological stress responses in adolescents, and differences in coping styles can cause differences in behaviors in adolescents. Coping style is a significant factor leading to smartphone addiction among adolescents. Problem-focused coping strategies indicate that coping behaviors directly target the source of stress and can prompt participants to use positive coping styles to deal with the adverse consequences of the pandemic; conversely, coping styles of avoidance, denial, and fantasy in dealing with stress make it a potentially strong risk factor for smartphone addiction ~~(Stahl GK, 2005; Duan L,2021)~~[65,66].

Limitations

Formatted: Font: (Default) Times New Roman, 12 pt, Not Italic

Formatted

This study had several limitations. The convenience sample limits the universality of the results. Factors such as family environment, personality traits, peer relationships, and sleep quality may also affect mobile phone addiction among adolescents. Therefore, future studies should examine whether the relationship between Chinese adolescent resilience, coping style, DASS, and mobile phone addiction will change over time.

Conclusions

This study explored the impact mechanism of the effect of resilience on mobile phone addiction among Chinese adolescents during a pandemic and flood.The main aim of the study was to establish if there was a relationship between resilience and mobile phone addiction in Chinese adolescents and if negative coping and DASS were a chain mediator of this relationship. The structural equation model was utilized to synchronously examine the individual and continuous mediating roles of coping styles and DASS. The results of This study results indicate that a negative relationship exists between resilience and mobile phone addiction in this population. In addition, stress, anxiety, depression, and coping style significantly influence the risk of adolescent mobile phone addiction and play an intermediary role in Chinese adolescent resilience and mobile phone addiction. These results indicate the importance of mobile phone addiction and the importance of resilience for adolescents.Other studies have also shown that smartphone addiction caused health problems ，such as eating, sleeping,and mood disorder[67]. The findings may also help educators and medical

Formatted: Normal, Space Before: 6 pt, After: 12 pt, Line spacing: Double

personnel distinguish between predictive factors for adolescent mobile phone addiction.~~;~~ T~~t~~hey could also be used to design an intervention to effectively treat and prevent mobile phone addiction in adolescents when dealing with future difficult and traumatic events. At the same time, it also indicates the importance of cultivating the psychological resilience of adolescents in coping with pressure and difficulties since having good psychological resilience can reduce depression, anxiety, and reduce mobile phone addiction.

Formatted: Font: (Default) Times New Roman, 12 pt, Font color: Auto, Pattern: Clear

Formatted: Font: 12 pt

Formatted: Font: (Default) Times New Roman, 12 pt, Font color: Auto, Pattern: Clear

Formatted: Font: (Default) Times New Roman, 12 pt, Font color: Auto

Formatted: Font: (Default) Times New Roman, 12 pt, Font color: Auto

Formatted: Normal (Web), Space Before: 0 pt, After: 0 pt, Line spacing: At least 12 pt, Pattern: Clear (Custom Color(RGB(252,253,254)))

List of abbreviations

DASS: depression, anxiety, and stress; MPAI: mobile phone addiction index; RSCA: the Resilience Scale for Chinese Adolescents ; SCSQ: the Simplified coping style questionnaire.

Acknowledgements

Not applicable.

References

1. Lin YH, Chang LR, Lee YH, Tseng HW, Kuo TB, Chen SH. Development and validation of the Smartphone Addiction Inventory (SPAI). PLoS One. 2014 ;9(6):e98312. doi: 10.1371/journal.pone.0098312.

~~1~~2. China Internet Network Information Center. https://www.cnnic.net.cn/hlwfzyj/hlwxzbg/hlwtjbg/202109/P020210915523670981527.pdf

~~2~~3. Patton GC, Sawyer SM, Santelli JS, et al. Our future: a Lancet commission on adolescent health and wellbeing.Lancet.2016;387:2423-2478.

Formatted: Font color: Auto

Formatted: None, Indent: Left: 0", Hanging: 0.25", Space Before: 6 pt, After: 6 pt

Formatted: Font: 12 pt, Not Bold

~~3~~4. Ozamiz-Etxebarria, N., Dosil-Santamaria, M., Picaza-Gorrochategui, M., & Idoiaga-Mondragon, N. Stress, anxiety, and depression levels in the initial stage of the COVID-19 outbreak in a population sample in the northern Spain. Cadernos de Saude Publica.2020;36(~~:~~4)~~:~~;~~,~~e00054020. ~~https://~~doi~~.~~:org/10.1590/0102-311X00054020

~~4~~5.Guang-Li Lu,Yue-Ming Ding,Yi-Ming Zhang, Hai-Tao Huang, Yi-Pei Liang, Chao-Ran Chen. The correlation between mobile phone addiction and coping style among Chinese adolescents: a meta-analysis. Child and Adolescent Psychiatry and Mental Health.2021;15:60 . ~~https://~~doi~~.~~:org/10.1186/s13034-021-00413-2

~~5~~6.The Tenth Press Conference of Henan Province Flood Control and Disaster Relief. Available online: http://www.henan.gov.cn/2021/08-02/2194036.html (accessed on 27 August 2021).

~~6~~7. Enoka Randeniya. Flooding disaster: The effect on the adolescents at Angoda RahulaCollege, Sri Lanka. Procedia Engineering.2018;723–728.

~~7~~8. Makwana N. Disaster and its impact on mental health: A narrative review. J Family Med Prim Care .2019;8:3090-5. ~~DOI~~doi:10.4103/jfmpc.jfmpc_893_19.

~~8. Satadeepa Som, Sunil Kumar Giriyappa Patil. Gender differences in coping behaviour among adolescents.International Journal of Psychology.2019;12,2: 39-42. http://www.medpulse.in~~

~~9. Breik, Wisam,Zaza, Haidar.Coping strategies adopted by adolescents: A comparative study in relation to gifted status, gender, and family size.Gifted Education International.2019;35,1:3-19. DOI:10.1177/0261429418824118.~~

9.Agnew, R. (1992). Foundation for a general strain theory of crime and delinquency.Criminology.1992;30(1): 47–88. doi.org/10.1111/j.1745-9125.1992.tb01093.x

10.Jun, S., & Choi, E. (2015). Academic stress and Internet addiction from general strain theory framework. Computers in Human Behavior.2015;49:282–287. doi:10.1016/j.chb.2015.03.001

11.Greco R, Curci A. Does the General Strain Theory Explain Gambling and Substance Use? J Gambl Stud. 2017 ;33(3):919-936. doi: 10.1007/s10899-016-9654-2.

12.Mubarak, A. and Quinn, S. . General strain theory of Internet addiction and deviant behaviour in social networking sites (SNS), Journal of Information, Communication and Ethics in Society. 2019;17(1):61-71. doi.org/10.1108/JICES-08-2016-0024

13.Kim, Chaeyoon & Kwak, Keumjoo & Kim, Yeonsoo. The Relationship between Stress and Smartphone Addiction among Adolescents: The Mediating Effect of Grit. 2021. doi: org/10.21203/rs.3.rs-159399/v1

Formatted: Indent: Left: 0", Hanging: 1 ch, First line: -1 ch

Formatted: Left, Indent: Left: 0", Hanging: 0.25", Space Before: 6 pt, After: 6 pt, Line spacing: single, Widow/Orphan control

Formatted: Font: Font color: Auto, Text Fill

Formatted: Font: Font color: Auto, Text Fill

Formatted: Indent: First line: 1 ch, No bullets or numbering, Tab stops: Not at 0.22"

Formatted: Indent: First line: 1 ch

Formatted: Indent: First line: 1 ch, No bullets or numbering, Tab stops: Not at 0.22"

Formatted: Indent: First line: 1 ch

Formatted: Indent: Left 1.14 ch, No bullets or numbering, Tab stops: Not at 0.22"

Formatted: Indent: First line: 1 ch

Formatted: Line spacing: single

Formatted: Indent: Left 1.14 ch, Line spacing: single, No bullets or numbering, Tab stops: Not at 0.22"

14.Tingting Gao, Jiaomeng Li, Han Zhang, Jinglei Gao, Yixi Kong, Yueyang Hu, Songli Mei.The influence of alexithymia on mobile phone addiction: The role of depression, anxiety and stress,Journal of Affective Disorders.2018;225:761-766. doi:org/10.1016/j.jad.2017.08.020.

15.Chih Hung Ko, Ju Yu Yen, Cheng Fang Yen, Cheng Sheng Chen, Chih Chi Weng, Cheng Chung Chen.The Association between Internet Addiction and Problematic Alcohol Use in Adolescents: The Problem Behavior Model.2008;11(5), 571-576. doi:org/10.1089/cpb.2007.0199

16.Makwana N. Disaster and its impact on mental health: A narrative review. J Family Med Prim Care. 2019 ;8(10):3090-3095. doi: 10.4103/jfmpc.jfmpc_893_19.

17.Ide-Okochi, A.; Samiso, T.;Kanamori, Y.; He, M.; Sakaguchi, M.;Fujimura, K. Depression, Insomnia,and Probable Post-Traumatic Stress Disorder among Survivors of the 2016 Kumamoto Earthquake and Related Factors during the Recovery Period Amidst the COVID-19 Pandemic. Int. J. Environ. Res. Public Health.2022;19:4403. doi:org/10.3390/ijerph19074403

18.Esterwood, E., Saeed, S.A. Past Epidemics, Natural Disasters, COVID19, and Mental Health: Learning from History as we Deal with the Present and Prepare for the Future. Psychiatr Q.2020;91:1121–1133. doi:org/10.1007/s11126-020-09808-4

19.Carli V, Durkee T, Wasserman D, Hadlaczky G, Despalins R, Kramarz E, et al. The association between pathological internet use and comorbid psychopathology: a systematic review. Psychopathology .2013;46:1-13.

20.Robert Fuchs, Maurya Glaude, Tonya Hansel, Joy Osofsky & Howard Osofsky. Adolescent risk substance use behavior, posttraumatic stress, depression, and resilience: Innovative considerations for disaster recovery, Substance Abuse.2021;42(3):358-365.doi: 10.1080/08897077.2020.1784357

21.Vella SL, Pai NB. A theoretical review of psychological resilience: Defining resilience and resilience research over the decades. Arch Med Health Sci 2019;7:233-9.doi:10.4103/amhs.amhs_119_19

22.Schriber, R. A. & Guyer, A. E. Adolescent neurobiological susceptibility to social context. Dev. Cogn. Neurosci.2016;19:1–18.

23.Harms, P. D., Brady, L., Wood, D., & Silard, A.. Resilience and well-being. In E. Diener, S.Oishi, & L. Tay (Eds.), Handbook of well-being. Salt Lake City, UT: DEF Publishers 2018.doi:nobascholar.com

24.Shen X. (2020) Is psychological resilience a protective factor between motivations and excessive smartphone use? Journal of Pacific Rim Psychology.2020; 14: e17. doi:org/10.1017/prp.2020.10

25.~~10.~~Davis SJ, Spillman S. Reasons for drug abstention: a study of drug use and resilience. J Psychoactive Drugs .2011;43:14-19.

26.Liu, Q.-Q., Zhou, Z.-K., Yang, X.-J., Kong, F.-C., Niu, G.-F., & Fan, C.-Y.. Mobile phone addiction and sleep quality among Chinese adolescents: A

Formatted: Line spacing: single

Formatted: Indent: Left 1.14 ch, Line spacing: single, No bullets or numbering, Tab stops: Not at 0.22"

Formatted: Indent: First line: 1 ch, Line spacing: single

Formatted: Line spacing: single

Formatted: Indent: Left 1.14 ch, Line spacing: single, No bullets or numbering, Tab stops: Not at 0.22"

Formatted: Indent: First line: 1 ch, Line spacing: single

Formatted: Line spacing: single

Formatted: Indent: First line: 1 ch, Line spacing: single, No bullets or numbering, Tab stops: Not at

Formatted: Line spacing: single

Formatted: Indent: Left 1.14 ch, Line spacing: single, No bullets or numbering, Tab stops: Not at 0.22"

Formatted: Line spacing: single

Formatted: Indent: Left 1.14 ch, Line spacing: single, No bullets or numbering, Tab stops: Not at 0.22"

Formatted: Indent: Left: 0", Hanging: 1 ch, First line: -1 ch

Formatted: Font: Font color: Auto

moderated mediation model. Computers in Human Behavior.2017; 72;108–114. doi:10.1016/j.chb.2017.02.042

27.Chen, L., Yan, Z., Tang, W., Yang, F., Xie, X., & He, J. . Mobile phone addiction levels and negative emotions among Chinese young adults: The mediating role of interpersonal problems. Computers in Human Behavior.2016;55;856-866.doi:10.1016/j.chb.2015.10.030

28.Dong, G. S., Park, Y., Min, K. K., & Park, J. . Mobile phone dependency and its impacts on adolescents' social and academic behaviors. Computers in Human Behavior.2016;63;282–292.doi:10.1016/j.chb.2016.05.026

~~11. Carli V, Durkee T, Wasserman D, Hadlaczky G, Despalins R, Kramarz E, et al. The association between pathological internet use and comorbid psychopathology: a systematic review. Psychopathology .2013;46:1-13.~~

~~12~~29. Lazarus, R. S., and Folkman, S. Stress, Appraisal, and Coping. New York,NY: Springer1984.

~~13~~30. Chapman, Paula L.; Mullis, Ronald L. Adolescent Coping Strategies and Self-Esteem. Child Study Journal.1999;29,1: 69-77.

~~14~~31. Orgilés M, Morales A, Delvecchio E,et al. Coping Behaviors and Psychological Disturbances in Youth Affected by the COVID-19 Health Crisis. Front.Psychol. 2021;12:565-657.doi:org/10.3389/fpsyg.2021.565657

~~15~~32.Fang Liu, Zhonghao Zhang, Liang Chen.Mediating effect of neuroticism and negative coping style in relation to childhood psychological maltreatment and smartphone addiction among college students in China.Child Abuse & Neglect.2020;106. ~~https://~~doi~~.~~:org/10.1016/j.chiabu.2020.104531.

~~16~~33.de Terte, I., & Stephens, C. Psychological resilience of workers in high- risk occupations (Editorial). Stress and Health: Journal of the International Society for the Investigation of Stress.2014;30,5: 353–355. ~~https://~~doi~~.~~:org/10.1002/smi.2627

~~17. Schriber, R. A. & Guyer, A. E. Adolescent neurobiological susceptibility to social context. Dev. Cogn. Neurosci.2016;19:1–18.~~

~~18. Southwick, S. M., Bonanno, G. A., Masten, A. S., Panter-Brick, C. & Yehuda, R. Resilience definitions, theory, and challenges: interdisciplinary perspectives.European Journal of Psychotraumatology .2014;5:25338.~~

~~http://dx.doi.org/10.3402/ejpt.v5.25338~~

~~19. Southwick, S. M. & Charney, D. S. The science of resilience: implications for the prevention and treatment of depression. Science .2012;338: 79–82.~~

~~20. Hu, T., Zhang, D. & Wang, J. A meta-analysis of the trait resilience and mental health. Pers. Individ. Dif.2015;76: 18–27 .~~

Formatted: Font: Font color: Auto

Formatted: Font: Font color: Auto

Formatted: Font: (Default) Times New Roman

Formatted: Font: (Default) Times New Roman

Formatted: Font: (Default) Times New Roman

Formatted: Font: (Default) Times New Roman

Formatted: Font: (Default) Times New Roman

Formatted: Font: (Default) Times New Roman

Formatted: Indent: Left: 0", First line: 0"

21. Malhi, G.S., Das, P., Bell, E. et al. Modelling resilience in adolescence and adversity: a novel framework to inform research and practice. Transl Psychiatry.2019;9:316. https://doi.org/10.1038/s41398-019-0651-y

2234.Li J, Chen Y-p, Zhang J, Lv M-m,Välimäki M, Li Y-f, Yang S-l, Tao Y-x,e B-y, Tan C-x and Zhang J-p.The Mediating Role of Resilience and Self-Esteem Between Life Events and Coping Styles Among Rural Left-Behind Adolescents in China: A Cross-Sectional Study.Front. Psychiatry .2020;11:560556.doi: 10.3389/fpsyt.2020.560556

2335. Wu Y, Sang Z, Zhang X-C, Margraf J.The Relationship Between Resilience and Mental Health in Chinese College Students: A Longitudinal Cross-Lagged Analysis. Front. Psychol.2020;11:108.doi: 10.3389/fpsyg.2020.00108

2436. Smida, M., Khoodoruth, M. A. S., Al-Nuaimi, S. K., et al. Coping strategies, optimism and resilience factors associated with mental health outcomes among medical residents exposed to coronavirus disease 2019 in Qatar. Brain and Behavior.2021;11:e2320. https://doi.:org/10.1002/brb3.2320

2537. Lovibond, P.F.; Lovibond, S.H. The structure of negative emotional states: Comparison of the Depression Anxiety Stress Scales (DASS) with the Beck Depression and Anxiety Inventories. *Behav. Res. Ther*.1995;*33*:335–343.

2638. Louis Leung. Linking psychological attributes to addiction and improper use of the mobile phone among adolescents in Hong Kong. Journal of Children and Media.2008;2(2):93-113. DOIdoi:10.1080/17482790802078565

2739. Yueqin Hu,Yiqun Gan. Development and validity of Resilience Scale for Chinese Adolescent. Acta Psychologica Sinica.2008;40(8)：902-912.

2840.Ya-Ning Xie. A preliminary study on the reliability and validity of the Simplified Coping Style questionnaire. Chinese Journal Clinical Psychology.1998;6(2):114-115.

2941.Baron,R.M.,Kenny,D.A.. The moderator–mediator variable distinction in social psychological research: Conceptual, strategic, and statistical considerations. Journal of Personality and Social Psychology.1986;51(6):1173-1182.

3042. Hu, L. T., and Bentler, P. M.. Cutoff criteria for fit indexes in covariance structure analysis: conventional criteria versus new alternatives. Struct. Equ. Model.1999; 6(1):1–55.

43.31.Kline RB. Principles and Practice of Structural Equation Modeling. 2nd ed. New York, NY: Guildford Press;2005.

32. Robertson, T.W.; Yan, Z.; Rapoza, K.A. Is Resilience a Protective Factor of Internet Addiction? Comput. Hum. Behav.2018;78:255–260. https://doi.org/10.1016/j.chb.2017.09.027

44.~~33~~.Kumpfe K.L. and Bluth B. Parent/child transactional processes predictive of resilience or vulnerability to “substance abuse disorders”. Substance Use & Misuse.2004;39: 671–698.

45~~34~~. Mark Griffiths. A ‘components’ model of addiction within a biopsychosocial framework. Journal of Substance Use.2005;10(4): 191–197.

46~~35~~. Lee Y.K., Chang C.T., Lin Y. and Cheng Z.H. The dark side of smartphone usage: Psychological traits, compulsive behavior and technostress.

Computers in Human Behavior, 2014； 31, 373–383.

47~~36~~. Hou X.L., Wang H.Z., Guo C., Gaskin J., Rost D.H. and Wang J.L.

Psychological resilience can help combat the effect of stress on problematic

social networking site usage. Personality & Individual Differences,2017； 109,

61–66.

48.Morales-Rodríguez FM, Martínez-Ramón JP, Méndez I and Ruiz-Esteban C .Stress, Coping, and Resilience Before and After COVID-19: A Predictive Model Based on Artificial Intelligence in the University Environment. Front. Psychol. 2021;12:647964. doi: 10.3389/fpsyg.2021.647964

49.Li J, Chen Y-p, Zhang J, Lv M-m, Välimäki M, Li Y-f, Yang S-l, Tao Y-x, Ye B-y, Tan C-x and Zhang J-p.The Mediating Role of Resilience and Self-Esteem Between Life Events and Coping Styles Among Rural Left-Behind Adolescents in China: A Cross-Sectional Study. Front. Psychiatry.2020;11:560556. doi: 10.3389/fpsyt.2020.560556

50.He A, Wang C, Hui Q. Loneliness and smartphone dependence: The role of negative coping. Chinese J Clin Psychol. 2018;26(6):1222 – 5.

51.Gao, T., Li, J., Zhang, H., Gao, J., Kong, Y., Hu, Y., & Mei, S. (2018). The influence of alexithymia on mobile phone addiction: The role of depression, anxiety and stress. Journal of Affective Disorders.2018; 225:761 – 766. doi:10.1016/j.jad.2017.08.020

52.Yang, X., Zhou, Z., Liu, Q. et al. Mobile Phone Addiction and Adolescents’ Anxiety and Depression: The Moderating Role of Mindfulness. J Child Fam Stud.2019;28:822–830. doi:org/10.1007/s10826-018-01323-2

53.Choksi S, Patel N. A Study to Find Out the Correlation of Mobile Phone Addiction with Anxiety, Depression, Stress and Sleep Quality in the College Students of Surat City. Int J Curr. 2021;13(08):137-142.

doi: http://dx.doi.org/10.31782/IJCRR.2021.13812

Formatted: Line spacing: single

Formatted: Font color: Auto

54.Lei, L. Y.-C., Ismail, M. A.-A., Mohammad, J. A.-M., & Yusoff, M. S. B. . The relationship of smartphone addiction with psychological distress and neuroticism among university medical students. BMC Psychology.2020;8(1):97. doi:10.1186/s40359-020-00466-6

55.Buabbas AJ, Hasan H, Buabbas MA.The associations between smart device use and psychological distress among secondary and high school students in Kuwait. PLoS ONE.2021;16(6): e0251479. doi:org/10.1371/journal. pone.0251479.

56.Stanković M, Nešić M. Association of internet addiction with depression, anxiety, stress, and the quality of sleep: Mediation analysis approach in Serbian medical students. Current Research in Behavioral Sciences.2022;3: 100071.

doi:org/10.1016/j.crbeha.2022.100071

~~37. Alan R, Guzel HS. The investigation of the relationship between smartphone addiction, and problem-solving skills and ways of coping with stress. Dusunen Adam The Journal of Psychiatry and Neurological Sciences .2020;33:244-253.~~

57~~38~~. Lu GL, Ding YM, Zhang YM, Huang HT, Liang YP, Chen CR. The correlation between mobile phone addiction and coping style among Chinese adolescents: a meta-analysis. Child Adolesc Psychiatry Ment Health.2021;15(1):60. doi: 10.1186/s13034-021-00413-2.

58~~39~~.Ozen S, Topcu M. The relationship of smartphone addiction with depression, obsession-compulsion, impulsivity, alexithymia among medical faculty students. Journal of Dependence .2017;18:16-24.

59~~40~~.Elhai JD, Tiamiyu MF, Weeks JW, Levine JC, Picard KJ, Hall BJ. Depression and emotion regulation predict objective smartphone use measured over one week. Pers Individ Dif .2018;133:21-28.doi:org/10.1016/j.paid.2017.04.051.

60~~41~~. Pera A . The Psychology of Addictive Smartphone Behavior in Young Adults: Problematic Use, Social Anxiety, and Depressive Stress. Front. Psychiatry. 2020;11:573473. doi: 10.3389/fpsyt.2020.573473

~~42~~61. Sonali Tanmay Choksi; Nipa Patel. A Study to Find Out the Correlation of Mobile Phone Addiction with Anxiety, Depression, Stress and Sleep Quality in the College Students of Surat City. Int J Cur Res Rev .2021;13(8):137-142.

~~D~~d~~OI~~oi: ~~http://dx.doi.~~org/10.31782/IJCRR.2021.13812.

~~43~~62. Al. Qudah, M.F.; Albursan,I.S.; Hammad, H.I.; Alzoubi, A.M.;Bakhiet, S.F.; Almanie, A.M.; Alenizi,S.S.; Aljomaa, S.S.; Al-Khadher, M.M.Anxiety about COVID-19 Infection,and Its Relation to Smartphone Addiction and Demographic Variables in Middle Eastern Countries. Int. J. Environ. Res. Public Health.2021; 18: 11016. ~~https://~~doi:~~.~~org/10.3390/ijerph182111016.

Formatted: Indent: Left: 0", First line: 0", Space After: 12 pt

Formatted: Font: (Default) Times New Roman, 12 pt, No underline, Font color: Accent 1

Formatted: Font: Font color: Accent 1

Formatted: Indent: Left: 0", First line: 1 ch

~~44~~63. Ahn, S. Y., Kim, Y. J. The influence of smart phone use and stress on quality of sleep among nursing students. Ind. J. Sci. Technol.2015;8:1–6. doi:10.17485/ijst%2F2015%2Fv8i35%2F85943

~~45~~64. Einar B. Internet Addiction, Psychological Distress, and Coping Responses Among Adolescents and Adults. CYBERPSYCHOLOGY, BEHAVIOR, AND SOCIAL NETWORKING.2017;20(5):296-304. ~~DOI~~doi: 10.1089/cyber.2016.0669

~~46~~65. Stahl GK, Caligiuri P. The effectiveness of expatriate coping strategies: the moderating role of cultural distance, position level, and time on the international assignment. J Appl Psychol.2005;90:603–15. doi: 10.1037/0021-9010.90.4.603

~~47~~66. Duan L, He J, Li M, Dai J, Zhou Y, Lai F and Zhu G . Based on a Decision Tree Model for Exploring the Risk Factors of Smartphone Addiction Among Children and Adolescents in China During the COVID-19 Pandemic. Front. Psychiatry. 2021;12:652356. doi: 10.3389/fpsyt.2021.652356

67.Napassphol Sinsomsack,Waiphot Kulachai.A study on the impacts of smartphone addiction. Proceedings of the 15th International Symposium on Management.2018;186:248-252.doi:10.2991/insyma-18.2018.61

Formatted: Line spacing: single

Field Code Changed

Supporting information

S1 Fig.

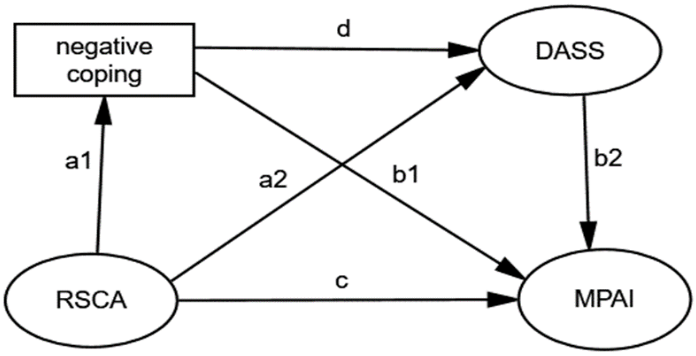

Figure 1 Hypothesized model

Notes: mobile phone addiction index (MPAI); depression, anxiety, and stress scale with 21 items (DASS-21); Resilience Scale for Chinese Adolescents (RSCA); Simplified coping style questionnaire (SCSQ).

S2 Fig.

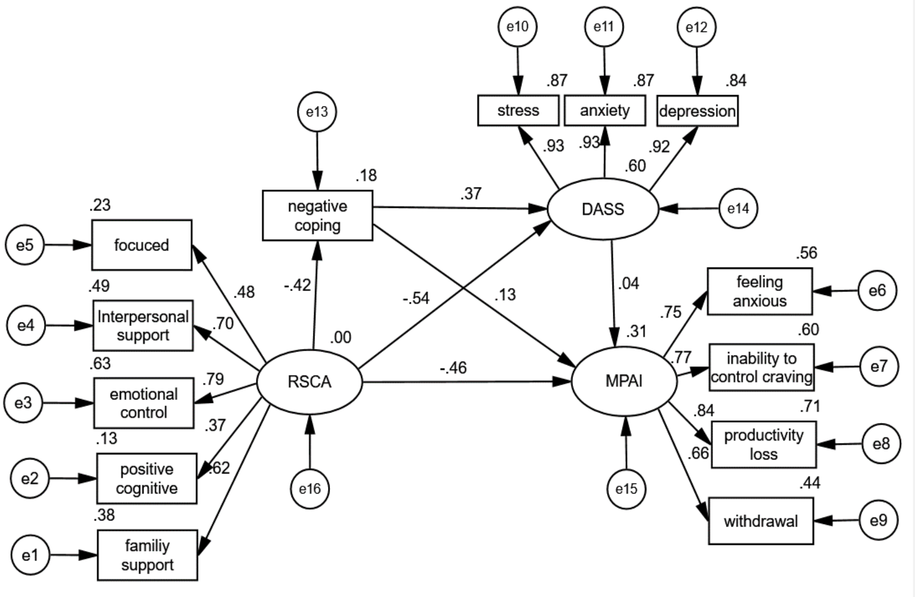

Figure 2 The standardized path coefficients in model testing

Formatted: Indent: Left: 0", First line: 0"

S1 Table.

Table 1: Demographic profiles and descriptive statistics of the participants.

**Gender**	Frequency	Percentage
boy	979	43.2
girl	1289	56.8
**One-child**		
yes	169	7.5
no	2099	92.5
**Birth order**		
1st	1048	46.2
2nd	1041	45.9
3rd	179	7.9
**Nationality**		
Han	2259	99.6
Hui	8	0.4
Miao	1	0.0
**Grade**		
middle school 7th	174	7.7
middle school 8th	634	28.0
middle school 9th	436	19.2
high school 1st	34	1.5
high school 2nd	471	20.8
high school 3rd	519	22.9
Total	2268	100.0

S2 Table.

	M	Std.Error of Mean	Frequency	Percentage
**Age**	14.90	2.58		
**MPAI Total**	39.57	13.82		
feeling anxious&lost	7.96	4.02		
inability to control craving	16.65	5.92		
productivity loss	7.64	3.28		
withdrawal	7.32	3.49		
**DASS-21 Total**	5.19	4.57		
**Stress**	7.40	5.07		
normal			2054.00	90.56
mild			124.00	5.47
moderate			76.00	3.35
severe			14.00	0.62
extremely severe			0.00	0.00
**Anxiety**	6.31	4.94		
normal			1583.00	69.80
mild			218.00	9.61
moderate			304.00	13.40
severe			103.00	4.54

extremely severe			60.00	2.65
**Depression**	6.58	5.09		
normal			1773.00	78.18
mild			258.00	11.38
moderate			179.00	7.89
severe			48.00	2.12
extremely severe			10.00	0.44
**SCSQ Total**	56.39	17.91		
positive coping	22.45	9.18		
negative coping	11.93	5.64		
**RSCA Total**	88.89	18.50		
focuced	16.24	4.88		
Interpersonal support	18.51	5.90		
emotional control	19.63	5.76		
Positive cognitive	14.30	3.78		
familiy support	16.77	4.50		

Table 2. Basic characteristics and measure scores.

S3 Table.

Table3: Correlation analysis of study variables

	**1**	**2**	**3**	**4**
**1.RSCA Total**	1			
**2.Negative coping**	-.317**	1		
**3.DASS-21 Total**	-.593**	.603**	1	
**4.MPAI Total**	-.405**	.322**	.448**	1

Note: ***P* < 0.01

S4 Table.

Table 4. Results of the structural model: tests of hypothesized associations between constructs.

			Estimate	S.E.	t-value	P
**negative coping**	<---	**RSCA**	-1.025	0.078	-13.216	***
**DASS**	<---	**negative coping**	0.292	0.017	16.871	***
**DASS**	<---	**RSCA**	-1.152	0.061	-18.992	***
**MPAI**	<---	**RSCA**	-0.836	0.07	-11.919	***
**MPAI**	<---	**negative coping**	0.09	0.017	5.355	***
**MPAI**	<---	**DASS**	-0.096	0.031	-3.092	0.002

Note: ***P < 0.01

S5 Table.

Table 5. Bootstrap truncated regression results.

				Bootstrapping				
	Product of coefficients			BC 95%CI		Percentile 95%CI		
Relationships	**point estimate**	***SE***	***Z***	**Lower**	**Upper**	**Lower**	**Upper**	***P***
				Indirect Effects				
**RSCA → negative coping → MPAI**	-0.092	0.018	-5.111	-0.125	-0.061	-0.125	-0.061	0
**RSCA → DASS → MPAI**	0.111	0.043	2.581	0.043	0.188	0.045	0.186	0.002

Formatted Table

**RSCA → negative coping → DASS→ MPAI**	0.029	0.011	2.636	0.012	0.048	0.012	0.048	0.002
**Total**	-0.789	0.052	-15.173	-0.889	-0.698	-0.89	-0.699	0

Dear editor,

Thank you very much for your letter and the reviewers' thoughtful, constructive comments on our manuscript submitted to PLOS ONE (ID: PONE-D-22-09528), entitled "Adolescent Resilience and Mobile Phone Addiction in Henan Province of China: Impacts of Chain Mediating, Coping Style".

We have carefully taken the comments into consideration and revised the manuscript accordingly. We hope that we have satisfactorily incorporated all the comments and suggestions. The following is the point-by-point response to editor and the reviewer. If any question arises, please let us know. Thank you very much for your consideration.

Sincerely,

Hong Juan Chang, PhD

Xinxiang Medical University

JinShui Avenue 601, Xinxiang, 453003, China

E-mail: changhj0812@126.com

1. Please ensure that your manuscript meets PLOS ONE's style requirements, including those for file naming.

Response

We appreciate the suggestion. We have checked the style and our style Consistent with PLOS ONE style templates.

2. Please amend your current ethics statement to address.

Response

We are sorry for causing this confusion. Informed written consent was obtained from the parents or guardians of each participant and add the same text to the "Ethics Statement" field of the submission form (via "Edit Submission").

3.We note that the grant information you provided in the 'Funding Information' and 'Financial Disclosure' sections do not match. When you resubmit, please ensure that you provide the correct grant numbers for the awards you received for your study in the 'Funding Information' section.

Response

Thank you for giving this suggestion. We have provided the correct grant numbers and information in the 'Funding Information' section.

4.In your Data Availability statement, you have not specified where the minimal data set underlying the results described in your manuscript can be found.

Response

We are sorry for our carelessness. We have amend the manuscript about

data statement section.

5. Please amend the manuscript submission data (via Edit Submission) to include

author Yan Yang, Shuangxi Guo, Xue Li, Shenhua Zhang

Response

We are sorry for our carelessness. We have amend the manuscript submission data (via Edit Submission) to include author Yan Yang, Shuangxi Guo, Xue Li, Shenhua Zhang.

6. Your ethics statement should only appear in the Methods section of your manuscript.

Response

Thanks for the reminder. We have revised the ethics statement in the Methods section.

7. Please upload a new copy of Figures 1 and 2 as the file does not open.

Response

Thanks for the reminder. We will upload a new copy of Figures 1 and 2 as the file.

Reviewer #1: This article may be corrected as follows :

1. In abstract to be improved to describe succinctly related to Introduction, Method, Result and Discussion, on a good abstract will also be seen what becomes novelty of the article.

2. The introduction must be explained two main aspects; there are aspects of the theory that is backgrounded and problems found in the field to look research gap from this article. Then at the end of the introduction will be able to see what is new from this study.

3. The methodology may need to be explained in more detail related to the research subject so that it becomes clear who the subject is.

4. The result and discussion should give the table and figure of results more in-depth, and you have not done.

5. Conclusions must undoubtedly be able to answer from the purpose of this study.

Response

Thank you for giving this suggestion.We revise the abstract section and introduction,and detail explain the subject,amend the result,discussion and conclusion.

Reviewer #2: Link between resilience and mobile phone addiction not made clear.

Resilience not well defined, how is it being applied and to what purpose?

What definition of mobile phone addiction? When does use become addiction? Why is this important?

AS well as consent from parents – did young people themselves get a chance to consent or at least give assent?

Last sentence page 3 seems out of place – reads like findings.

Greater focus on the impact of and recovery from crisis such as covid, on mobile phone use, resilience and mental and social, wellbeing would make a clearer rationale for the study.

The impact of covid could have been considered beyond the stress and anxiety it caused. It is natural to expect through lockdowns and social restrictions that adolescents would have become more reliant on mobile phones. The mention of covid here feels somewhat tokenistic and not very well thought through.

Greater explanation of methodology and methods would make it clearer why and how this research was done the way it was.

Discussion of the findings limits the usefulness of the research. It could be made much ore clear what the links are between main components of the research and the future implications of this for Chinese adolescents.

Some written English and referencing issues to be corrected.

Response

Thank you for giving this comments. We have remend the introduction of this article to emphasize definition of Resilience and mobile phone addiction,and explains the application of resilience in many studies.We explain the harm of mobile phone addiction to adolescents.The study complied with ethical requirements and was carried out on the basis of the consent of the parents or guardians of minors and adolescent themselves.We also revise the methods ,result ,discussion and conclusion section, the written English and referencing are corrected in the article.
