# Supplementary material for: Adolescent resilience and mobile phone addiction in Henan Province of China: Impacts of chain mediating, coping style
Source: PLoS One. 2022 Dec 27;17(12):e0278182. doi: 10.1371/journal.pone.0278182 (PMC9794036; doi:10.1371/journal.pone.0278182)
Supplement: Attachment — Submitted filename: Response to Reviewers.docx [file pone.0278182.s003.docx]

Dear editor,

Thank you very much for your letter and the reviewers’ thoughtful, constructive comments on our manuscript submitted to PLOS ONE (ID: PONE-D-22-09528), entitled “Adolescent Resilience and Mobile Phone Addiction in Henan Province of China: Impacts of Chain Mediating, Coping Style”.

We have carefully taken the comments into consideration and revised the manuscript accordingly. We hope that we have satisfactorily incorporated all the comments and suggestions. The following is the point-by-point response to editor and the reviewer. If any question arises, please let us know. Thank you very much for your consideration.

Sincerely,

*Hong Juan Chang*, PhD

Xinxiang Medical University

JinShui Avenue 601, Xinxiang, 453003, China

E-mail: changhj0812@126.com

1. Please ensure that your manuscript meets PLOS ONE's style requirements, including those for file naming.

***Response***

*We appreciate the suggestion. We have checked the* *style and our style Consistent with PLOS ONE style templates.*

2. Please amend your current ethics statement to address.

***Response***

*We are sorry for causing this confusion. Informed written consent was obtained from the parents or guardians of each participant and add the same text to the “Ethics Statement” field of the submission form (via “Edit Submission”).*

3.We note that the grant information you provided in the ‘Funding Information’ and ‘Financial Disclosure’ sections do not match. When you resubmit, please ensure that you provide the correct grant numbers for the awards you received for your study in the ‘Funding Information’ section.

***Response***

*Thank you for giving this suggestion. We have provided the correct grant numbers and information in the‘Funding Information’section.*

4.In your Data Availability statement, you have not specified where the minimal data set underlying the results described in your manuscript can be found.

***Response***

*We are sorry for our carelessness. We have amend the manuscript about data statement section.*

5. Please amend the manuscript submission data (via Edit Submission) to include author Yan Yang, Shuangxi Guo, Xue Li, Shenhua Zhang

***Response***

*We are sorry for our carelessness. We have amend the manuscript submission data (via Edit Submission) to include author Yan Yang, Shuangxi Guo, Xue Li, Shenhua Zhang.*

6. Your ethics statement should only appear in the Methods section of your manuscript.

***Response***

*Thanks for the reminder. We have revised the ethics statement in the Methods section.*

7. Please upload a new copy of Figures 1 and 2 as the file does not open.

***Response***

*Thanks for the reminder. We will upload a new copy of Figures 1 and 2 as the file.*

Reviewer #1: This article may be corrected as follows :
1. In abstract to be improved to describe succinctly related to Introduction, Method, Result and Discussion, on a good abstract will also be seen what becomes novelty of the article.
2. The introduction must be explained two main aspects; there are aspects of the theory that is backgrounded and problems found in the field to look research gap from this article. Then at the end of the introduction will be able to see what is new from this study.
3. The methodology may need to be explained in more detail related to the research subject so that it becomes clear who the subject is.
4. The result and discussion should give the table and figure of results more in-depth, and you have not done.
5. Conclusions must undoubtedly be able to answer from the purpose of this study.

***Response***

*Thank you for giving this suggestion.We revise the abstract section and introduction,and detail explain the subject,amend the result,discussion and conclusion.*

Reviewer #2: Link between resilience and mobile phone addiction not made clear.
Resilience not well defined, how is it being applied and to what purpose?
What definition of mobile phone addiction? When does use become addiction? Why is this important?
AS well as consent from parents – did young people themselves get a chance to consent or at least give assent?
Last sentence page 3 seems out of place – reads like findings.
Greater focus on the impact of and recovery from crisis such as covid, on mobile phone use, resilience and mental and social, wellbeing would make a clearer rationale for the study.
The impact of covid could have been considered beyond the stress and anxiety it caused. It is natural to expect through lockdowns and social restrictions that adolescents would have become more reliant on mobile phones. The mention of covid here feels somewhat tokenistic and not very well thought through.
Greater explanation of methodology and methods would make it clearer why and how this research was done the way it was.
Discussion of the findings limits the usefulness of the research. It could be made much ore clear what the links are between main components of the research and the future implications of this for Chinese adolescents.
Some written English and referencing issues to be corrected.

***Response***

*Thank you for giving this comments. We have remend the introduction of this article to emphasize definition of Resilience and mobile phone addiction,and explains the application of resilience in many studies.We explain the harm of mobile phone addiction to adolescents.The study complied with ethical requirements and was carried out on the basis of the consent of the parents or guardians of minors and adolescent themselves.We also revise the methods ,result ,discussion and conclusion section, the written English and referencing are corrected in the article.*
